# SARS-CoV-2 spike protein induces endothelial inflammation via ACE2 independently of viral replication

**DOI:** 10.1038/s41598-023-41115-3

**Published:** 2023-08-28

**Authors:** Augusto C. Montezano, Livia L. Camargo, Sheon Mary, Karla B Neves, Francisco J Rios, Ross Stein, Rheure A. Lopes, Wendy Beattie, Jacqueline Thomson, Vanessa Herder, Agnieszka M. Szemiel, Steven McFarlane, Massimo Palmarini, Rhian M. Touyz

**Affiliations:** 1 Research Institute of the McGill University Health Centre (RI-MUHC), Site Glen-Block E-Office: E01.3362, 1001, Boul. Decarie, Montreal, QC H4A3J1 Canada; 2 School of Cardiovascular and Metabolic Health, University of Glasgow, Glasgow, UK; 3 Strathclyde Institute of Pharmacy and Biomedical Sciences, University of Strathclyde, Glasgow, UK; 4 MRC Centre for Virus Research, University of Glasgow, Glasgow, UK; 5 McGill University, Montreal, Canada

**Keywords:** Cardiovascular diseases, Hypertension, Mechanisms of disease

## Abstract

COVID-19, caused by SARS-CoV-2, is a respiratory disease associated with inflammation and endotheliitis. Mechanisms underling inflammatory processes are unclear, but angiotensin converting enzyme 2 (ACE2), the receptor which binds the spike protein of SARS-CoV-2 may be important. Here we investigated whether spike protein binding to ACE2 induces inflammation in endothelial cells and determined the role of ACE2 in this process. Human endothelial cells were exposed to SARS-CoV-2 spike protein, S1 subunit (rS1p) and pro-inflammatory signaling and inflammatory mediators assessed. ACE2 was modulated pharmacologically and by siRNA. Endothelial cells were also exposed to SARS-CoV-2. rSP1 increased production of IL-6, MCP-1, ICAM-1 and PAI-1, and induced NFkB activation via ACE2 in endothelial cells. rS1p increased microparticle formation, a functional marker of endothelial injury. ACE2 interacting proteins involved in inflammation and RNA biology were identified in rS1p-treated cells. Neither ACE2 expression nor ACE2 enzymatic function were affected by rSP1. Endothelial cells exposed to SARS-CoV-2 virus did not exhibit viral replication. We demonstrate that rSP1 induces endothelial inflammation via ACE2 through processes that are independent of ACE2 enzymatic activity and viral replication. We define a novel role for ACE2 in COVID-19- associated endotheliitis.

## Introduction

COVID-19 is caused by SARS-CoV-2 and was designated as a pandemic in March 2020. The health and socio-economic impact of COVID-19 has been catastrophic worldwide. While COVID-19 is predominantly a respiratory disease, growing evidence shows that patients with pre-existing cardiovascular disease (CVD) are at higher risk of developing more severe COVID-19, while post-COVID-19 infection, individuals may be prone to CVD^1–3^. Similarly, to SARS-CoV, SARS-CoV-2 enters the cell^4^ via binding to the integral membrane glycoprotein, angiotensin-converting enzyme 2 (ACE2). SARS-CoV-2 entry mechanism is mediated by the SPIKE protein (S protein) on the viral coat, which is comprised of two subunits: the S1 subunit containing the receptor binding domain (RBD) to ACE2, and the S2 subunit, responsible for membrane fusion. When SARS-CoV-2 infects the cells, the virus binds to ACE2 using its S1 subunit, permitting the S2 subunit to undergo cleavage by the host transmembrane serine protease 2 (TMPRSS2), leading to fusion of the viral envelope and cell membrane, and subsequent internalisation of the virus, as well as the bound ACE2, by endocytosis^5, 6^.

In the cardiovascular system, angiotensin II (Ang II), the primary effector peptide of the renin–angiotensin–aldosterone system (RAAS), acts on the AT1 receptor (AT1R) to induce deleterious effects such as vasoconstriction, inflammation, and cell hypertrophy^7^. ACE2 catalyzes Ang II into angiotensin-(1–7) (Ang-(1–7)), which in turn counteracts the injurious effects of Ang II by inducing vasodilation, anti-inflammatory, and anti-fibrotic actions^8^. The cardiovascular systemic effects of COVID-19 could be explained by the virus interactions with ACE2. ‘Hijacking’ of ACE2 by the viral spike and its internalization, may lead to a shift from the protective to the deleterious axis of the RAAS, contributing to the vascular complications observed during COVID-19, such as endothelial cell inflammation^9–11^. Endotheliitis has been considered as an important component of ‘long’ COVID-19^11^.

In addition to its protective role in the cardiovascular system, ACE2 is a chaperone protein for the amino acid transporter B0AT1 (SLC6A19) involved in its membrane anchorage^12^, independently of enzymatic function. Considering the relationship between SARS-CoV-2 spike and ACE2, it is unclear whether this interaction is merely a mechanism of infection or whether spike:ACE2 interaction induces injurious vascular signalling and contributes to cardiovascular damage associated with COVID-19. In this study we hypothesised that SARS-CoV-2 via ACE2 has inflammatory effects, independent of dysregulation of enzymatic function and viral infection of endothelial cells.

## Results

### SARS-CoV-2 recombinant S1 protein induces endothelial inflammation and cell damage independently of viral infection

**Figure 1:**
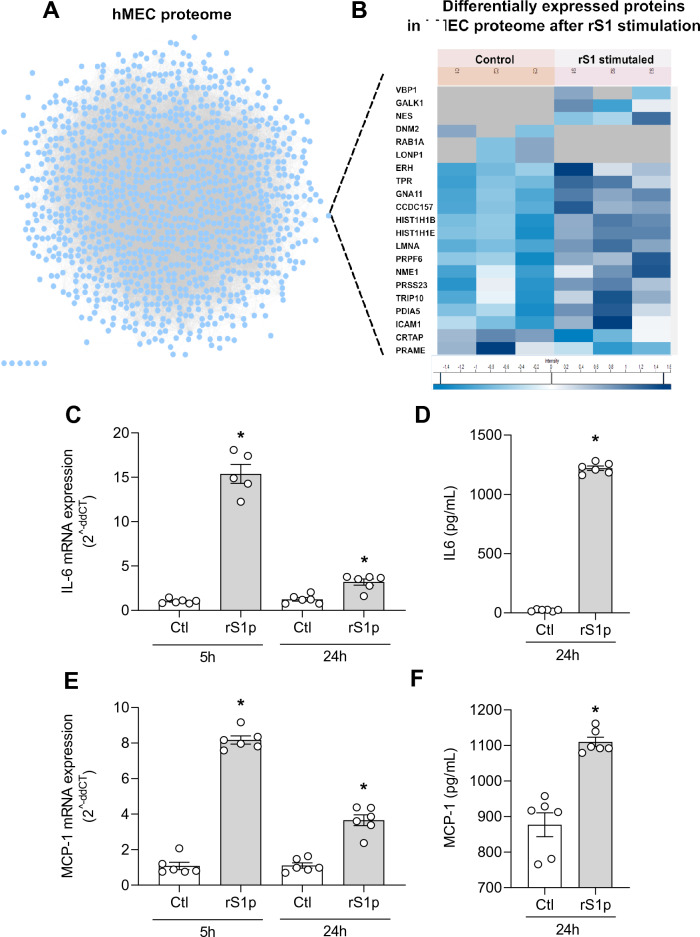
rS1p induces endothelial cell inflammation and injury. Proteomic analysis of rS1p-treated endothelial cells. (**A**) The figure represents 1150 hMEC protein interactome created by STRING. Each circular dot (blue, pink) represents the node (protein) and the grey lines the edges. (**B**) represents the heat map of differentially expressed proteins after 24 h of recombinant S1 spike protein (rS1p). Shades of blue represent the z-score intensities (scale below the heat map). Grey shade represents missing intensities. Human endothelial cells (hMEC) were stimulated with rS1p (0.66 μg/mL) for 5 h and 24 h for assessment of IL-6 (**C**) and MCP-1 (**D**) mRNA expression; IL-6 (**E**) and MCP-1 (**F**) production. Data are expressed as ± SEM; *p < 0.05 control (Ctl) (non-stimulated cells) vs. rS1p stimulated cells after student’s t-test.

To study the global effect of SARS-CoV-2 spike protein on endothelial cell protein expression, we assessed the proteome of hMEC after 24 h incubation with rS1p. After filtering the data for contaminants, reverse sequence, only identified by site, at least one unique peptide and 1% peptide and protein false discovery rate (FDR), a total of 1150 proteins were identified (Fig. 1A, Supplementary Data File). Biological process enrichment showed proteins from major signalling pathways and cellular processes, such as translational, virus transcription, cell–cell adhesion, NFκB signalling, oxidation–reduction process, MAPK cascade, T-cell receptor signalling, suggesting that rS1p induced endothelial cell inflammation and proliferation (Supplementary Data File). 21 proteins were found to be differentially expressed (Fold change > 2, p < 0.05) of which 5 were upregulated and 16 were downregulated after rS1p incubation (Fig. 1F, Supplementary Data File). Only 1.8% of the identified proteome was differentially expressed. These differentially expressed proteins (DEP) did not belong to any specific enriched gene ontology for molecular function and cellular components. However, they were closely linked to many processes associated with inflammation, including cell proliferation (NES, NME1, PRAME), endocytosis (NME1, RAB1A, DNM2, TRIP10), protein folding (VBP1, CRTAP, PDIA5), cell adhesion (RAB1A, DNM2, ICAM1), proteolysis (LONP1, PRSS23) and transcription (TPR, PRPF6, HIST1H1B, HIST1H1E, PRAME, DNME2). VBP1, GALK1, and NES were detected only in rS1 stimulated samples, while DNM2, RAB1A and LONP1 were detected only in control samples. ICAM1 a known cell adhesion molecule involved in inflammation was found to be upregulated in rS1-treated cells (Fig. 1B).

We further characterized rS1p pro-inflammatory responses in hMECs by measuring expression profiles and production of pro-inflammatory mediators. rS1p increased mRNA expression levels and release of the cytokine IL-6 (Fig. 1C and D) and the chemokine MCP-1 (Fig. 1E and F) in human endothelial cells. In addition, hMEC exposed to rS1p had higher gene levels of TNFα (Supplementary Fig. S2A), VCAM-1 (Supplementary Fig. S2B), PAI-1 (Supplementary Fig. S2C); while a decrease in mRNA expression of thrombin (Supplementary Fig. S2D) and angiopoietin-2 (Supplementary Fig. S2E) was observed. We did not observe any changes in gene expression of TGFβ (Supplementary Fig. S3A) and preproET-1 (Supplementary Fig. S3B).

**Figure 2:**
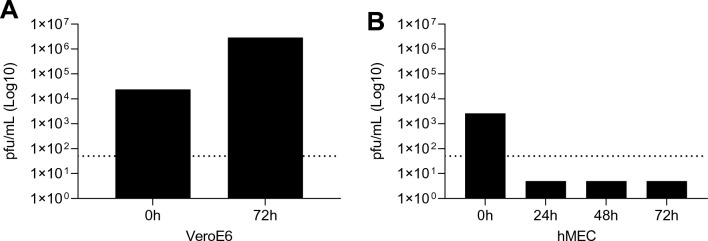
SARS-CoV-2 does not replicate in human endothelial cells but pseudovirus induces cell inflammation. SARS-CoV2 England-02 virus replication in hMECs. VeroE6 (**A**) and hMECs (**B**) were exposed to SARS-CoV-2 England-02 virus and virus plaques were measured after 72 h for VeroE6 cells and after 24 h, 48 h and 72 h for hMECs after incubation. The dotted line represents the lower limit of detection at 50 pfu/mL (n = 4–5). Data are expressed as ± SEM; *p < 0.05 negative vs. SARS-CoV-2 pseudotype virus exposed cells after 1-way ANOVA followed by Tukey’s post-hoc test.

To verify whether inflammation is associated with virus replication, we examined whether SARS-CoV2 virus replicates in hMEC. As shown in Fig. 2, hMECs cells were infected in 12-well plates with 10^5^ PFU per well and compared to Vero E6 cells, used as positive controls. Human MEC supernatants were then collected at 24-, 48-, and 72-h post-infection. We did not observe virus replication in hMECs, whereas in Vero E6 cells we detected titres of 10^6^ PFU per ml at 72 h post-infection (Fig. 2A and B).

**Figure 3:**
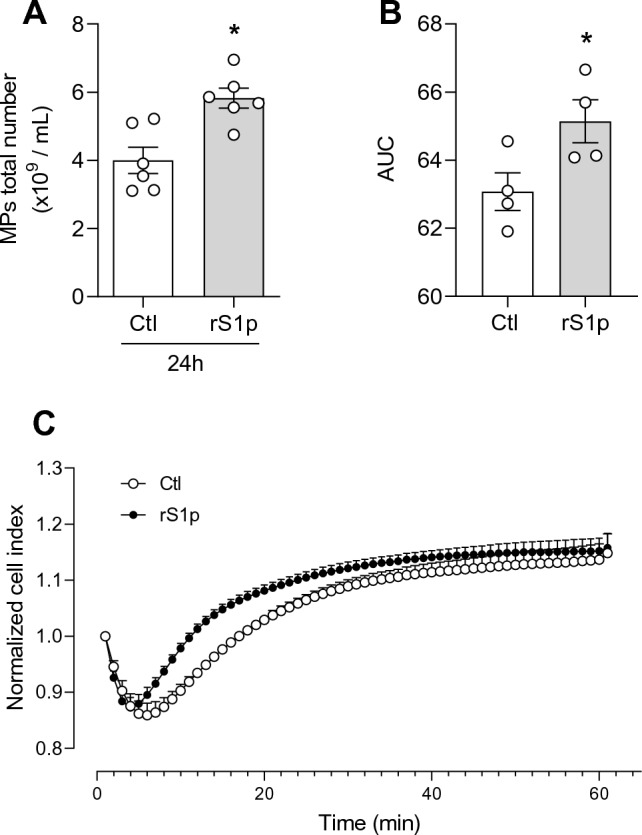
Endothelial cell microparticle formation and proliferation in response to rSP1. Human endothelial cells were stimulated with rS1p (0.66 μg/mL) for 5 h and 24 h for assessment of functional changes. Endothelial microparticle formation after 24 h exposure to rSP1 (**A**). Endothelial cell proliferation in response to rSP1 expressed as the cumulative area under the curve (AUC) (**B**) and the cell index/minutes of treatment with rS1p from xCELLigence studies (**C**). Data are expressed as ± SEM; *p < 0.05 control (Ctl) (non-stimulated cells) vs. rS1p stimulated cells after student’s t-test.

As indices of functional responses, we assessed the generation of microparticles and cell proliferation. Microparticles formation, a marker of endothelial cell injury, was increased by rS1p (10 min–24 h) (Fig. 3A and Supplementary Fig. S2F). Moreover, rS1p augmented endothelial cell proliferation due to increased cell impedance (Fig. 3B and C). Stimulation of hMEC with rS1p did not induce changes in NADPH-induced superoxide formation or H_2_O_2_ production (Supplementary Fig. S4A,B), as well as gene expression of antioxidant enzymes such as SOD1, catalase, glutathione peroxidase, peroxiredoxin, heme-oxygenase-1 and thioredoxin (Supplementary Fig. S5A–F).

The injurious actions of rS1p were not specific to human microvascular cells, as we observed similar pro-inflammatory profile in other endothelial cells from different vascular beds. rS1p increased IL-6 and MCP-1 mRNA levels and production in human lymphatic endothelial cells (hLEC) (Supplementary Fig. S6A–D), human aortic endothelial cells (hAEC) (Supplementary Fig. S7A–D) and human pulmonary artery endothelial cells (hPEC) (Supplementary Fig. S8A–D). Not all effects were similar between different cell lines, where microparticle formation was only increased by rS1p in hLECs (Supplementary Fig. S6E,F) and not in hAEC (Supplementary Fig. S7E,F) and hPEC (Supplementary Fig. S8E,F). ROS production, but not H_2_O_2_ levels, was increased in hLECs at 30 min (Supplementary Fig. S6G,H) after rS1p stimulation, but not in hAECs (Supplementary Fig. S7G,H) and hPECs (Supplementary Fig. S8G,H).

### ACE2 contributes to SARS-CoV-2 recombinant S1 protein-induced inflammation

**Figure 4:**
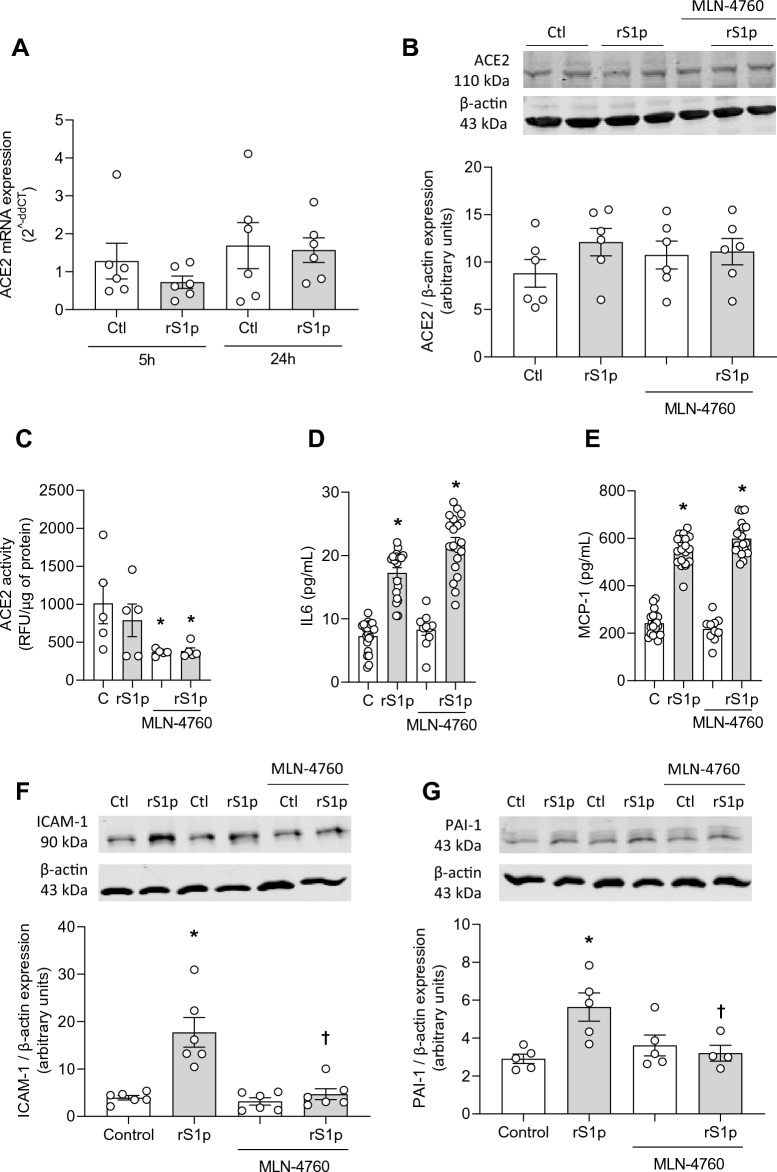
rS1p pro-inflammatory effects in human endothelial cells are mediated via ACE2. Human endothelial cells (hMEC) were stimulated with rS1p (0.66 μg/mL) for 5 h and 24 h for assessment of ACE2 mRNA levels (**A**), protein expression (**B**) and activity (**C**) in the presence or absence of MLN-4760 (440 pmol), an ACE2 inhibitor. IL-6 (**D**) and MCP-1 (**E**) production; and ICAM-1 (**F**) and PAI-1 (**G**) protein expression were also assessed after rS1p stimulation in the presence or absence of MLN-4760 (n = 5–20). Data are expressed as ± SEM; *p < 0.05 control (Ctl) (non-stimulated cells) vs. rS1p stimulated cells; ^†^p < 0.05 rS1p stimulated cells vs. rS1p stimulated cells treated with MLN-4760 after 1-way ANOVA followed by Tukey’s post-hoc test. Original blots are presented in Supplementary Figs. S13 and S14.

To better understand the putative role of ACE2 in rS1p inflammatory effects, we assessed whether ACE2 is regulated by rS1p in hMECs. rS1p did not alter ACE2 mRNA (Fig. 4A) and protein (Fig. 4B) expression levels, or ACE2 activity (Fig. 4C). In follow up studies, we used MLN-4760, an ACE2 inhibitor, to evaluate whether ACE2 inhibition potentiates or blocks rS1p-induced inflammation. MLN-4760 did not alter ACE2 protein expression (Fig. 4B) but reduced ACE2 activity to the same extent in cells exposed or not to rS1p (Fig. 4C). rS1p-induced IL-6 (Fig. 4D) and MCP-1 (Fig. 4E) production were not influenced by ACE2 inhibition. Similar results were observed at the gene level, where MLN-4760 did not inhibit rS1p-induced increase in mRNA expression in IL-6 and MCP-1 (Supplementary Fig. S9A,B). ACE2 inhibition blocked the increase in ICAM-1 (Fig. 4F) and PAI-1 (Fig. 4G) protein expression; despite not altering rS1p effects on PAI-1 mRNA levels (Supplementary Fig. S9C). VCAM-1 gene expression increase induced by rS1p in hMECs was blocked by MLN-4760 (Supplementary Fig. S9D). ACE2 enzymatic function activation by diminazine aceturate (DIZE) did not influence rS1p-induced increase in IL-6, MCP-1 and TNFα in hMECs (Supplementary Fig. S10A–C).

To gain some insights on how ACE2 associates with signaling pathways, we investigated whether ACE2 interacts with chaperone proteins by subjecting hMEC to co-immunoprecipitation with anti-ACE2 coupled to mass spectrometry protein identification. This led to identification of 216 proteins (filtered with ≥ 1 unique peptide, 1% FDR) along with ACE-2, which were identified by mass spectrometry with 11 peptides in both control and rS1 stimulated cell lysate and western blot (supplementary data file; Supplementary Fig. S11A,B). The 216 ACE2 interacting proteins belonged to various enriched biological processes especially linked to RNA cell biology, viral responses and inflammation, as detailed in supplementary Fig. S11C.

**Figure 5:**
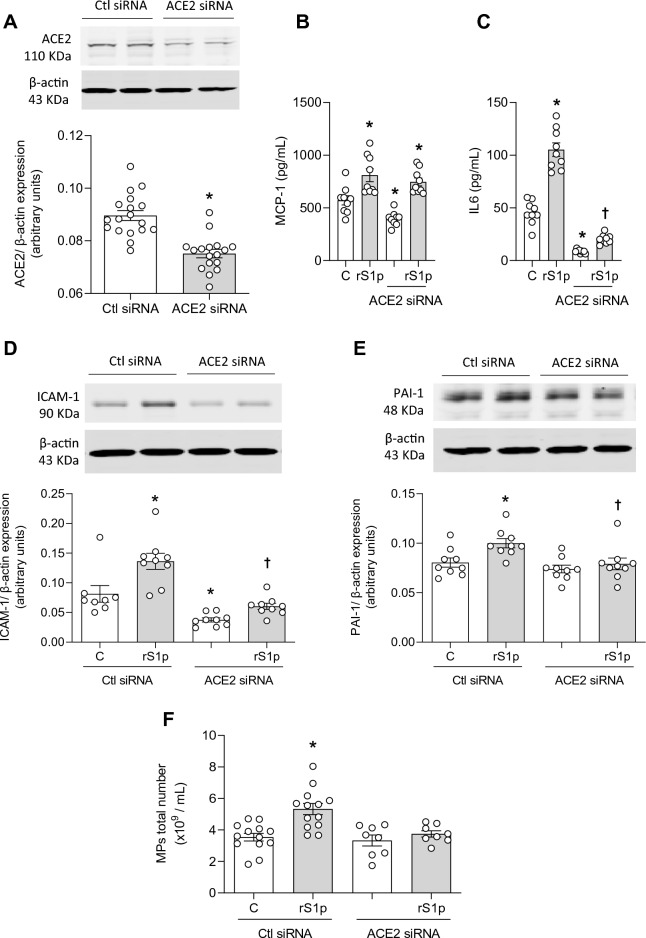
ACE2 siRNA blocks rS1p pro-inflammatory effects in human endothelial cells. (**A**) ACE2 siRNA efficiency. Human endothelial cells (hMEC) were stimulated with rS1p (0.66 μg/mL) for 24 h for assessment of IL-6 (**B**) and MCP-1 (**C**) production, ICAM-1 (**D**) and PAI-1 (**E**) protein expression, and microparticles formation (**F**) in the presence or absence of ACE2 siRNA (n = 9). Data are expressed as ± SEM; *p < 0.05 control (Ctl) (non-stimulated cells) vs. rS1p stimulated cells; ^†^p < 0.05 rS1p stimulated cells vs. rS1p stimulated cells treated with ACE2 siRNA after student’s t-test (**A**) or 1-way ANOVA followed by Tukey’s post-hoc test. Original blots are presented in Supplementary Figs. S15, S16 and S17.

**Figure 6:**
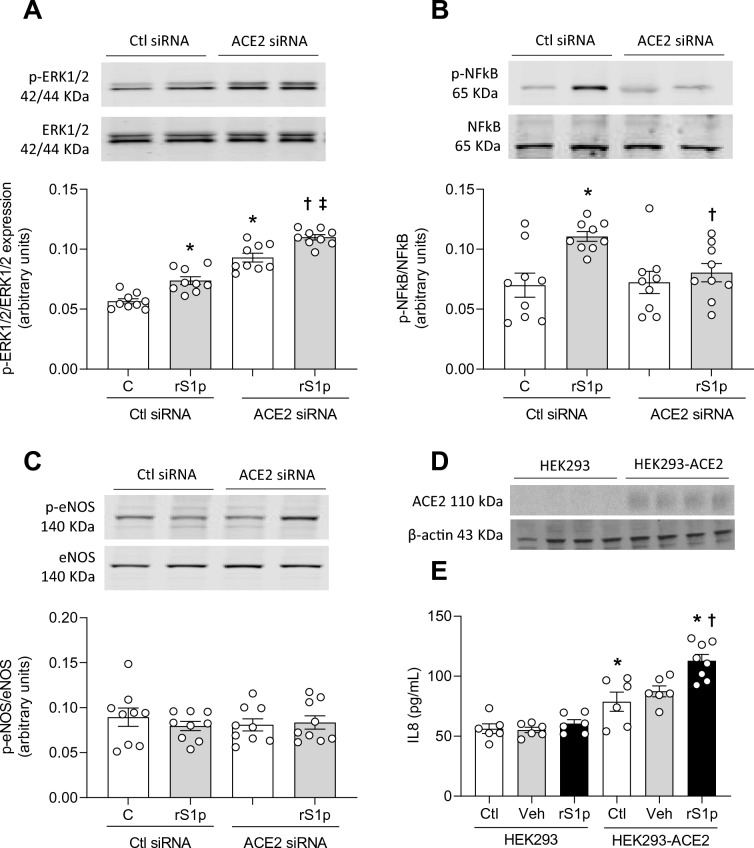
ACE2 siRNA blocks pro-inflammatory signalling activated by rS1p in human endothelial cells. Human endothelial cells (hMEC) were stimulated with rS1p (0.66 μg/mL) for 10 min for assessment of ERK1/2 (**A**), NFκB (**B**) and eNOS (**C**) phosphorylation in the presence or absence of ACE2 siRNA (n = 9). Human embryonic kidney (HEK) cells expressing (HEK293-ACE2) or not (HEK293) ACE2 (**D**) were stimulated with rS1p (0.66 μg/mL) for 24 for assessment of IL-8 production (**E**). Data are expressed as ± SEM; *p < 0.05 control (Ctl) (non-stimulated cells) vs. rS1p stimulated cells or HEK293 vs. HEK293-ACE2 non-stimulated cells; ^†^p < 0.05 rS1p stimulated cells vs. rS1p stimulated cells treated with ACE2 siRNA or rS1p stimulated HEK293 cells vs. rS1p stimulated HEK293-ACE2 cells; ^‡^p < 0.05 ACE siRNA vs. rS1p stimulated cells treated with ACE2 siRNA after 1-way ANOVA followed by Tukey’s post-hoc test. Original blots are presented in Supplementary Figs. S18, S19, S20 and S21.

Figure 5A demonstrates the efficacy of our ACE2 knockdown protocol, where ACE2 protein expression was decreased by siRNA. A decrease in ACE2 expression did not influence rS1p-induced MCP-1 production (Fig. 5B) but decreased its basal levels and rS1p-induced IL-6 release (Fig. 5C) and ICAM-1 expression (Fig. 5D). ACE2 siRNA also decreased PAI-1 expression (Fig. 5E) and microparticle formation (Fig. 5F) induced by rS1p in hMECs. In addition, neither rS1p nor ACE2 siRNA alter ET-1 (Supplementary Fig. S12A) and angiopoietin-2 (Supplementary Fig. S12B) production. Pro-inflammatory signalling pathways were assessed in hMECs after rS1p before and after ACE2 knockdown. ERK1/2 activation was increased after ACE2 siRNA with no changes on rS1p-induced effects (Fig. 6A), while rS1p-induced NFκB phosphorylation was blocked by ACE2 siRNA (Fig. 6B). No changes were observed in the phosphorylation of eNOS activation site (Ser1177) (Fig. 6C).

To further confirm the interdependence of SARS-CoV-2 spike protein and ACE2 in inflammatory responses, we exposed ACE2-expressing HEK cells expressing to rS1p. Compared to control HEK cells, that do not express ACE2 (Fig. 6D), rS1p increased IL-8 only in HEK293-ACE2, while no changes were observed when cells were exposed to only medium or vehicle (Fig. 6E).

## Discussion

COVID-19 is a viral infection of the respiratory system^5, 13,14^. It is also a systemic disease predisposing to cardiovascular sequelae such as thrombosis, hypertension, myocarditis, heart failure, arrhythmias, and acute kidney injury^2^. The association between between COVID-19 and cardiovascular comorbidities may relate to the pleiotropic role of ACE2^1, 9^. ACE2 is a key enzyme in RAAS homeostasis, where it regulates the degradation of the vasoconstrictor Ang II and formation of the vasodilator Ang(1–7)^15^, which in the context of SARS-CoV-2 infection has not been fully elucidated. Our study demonstrates that ACE2 plays a key role in inducing endothelial cell inflammation by SARS-CoV-2, independent of its enzymatic activity and viral replication. We present novel findings that SARS-CoV-2/ACE2 may be an important signalling effector that controls cellular responses related to inflammation, a hallmark of COVID-19^11, 16^.

Tissue damage commonly observed in COVID-19 is due to activation of immune pathways, affecting vascular cells such as endothelial cells and leading to loss of integrity, vascular dysfunction, and inflammation^17–21^. Endotheliitis has been described in COVID-19 patients, and molecular mechanisms involved are still under debate. Here we demonstrate that SARS-CoV-2 is a potent inducer of pro-inflammatory molecules, such as cytokines (IL-6, TNFα), chemokines (MCP-1), PAI-1 and adhesion molecules (ICAM-1, VCAM-1), and that it activates pro-inflammatory signalling through NFκB and ERK1/2 in endothelial cells. These effects were associated with microparticle formation, an important biomarker of endothelial cell damage and apoptosis^22^ as well as cell activation and proliferation. Clinical studies showed that raised serum levels of IL-6 and TNF-α are important prognostic factors in patients with COVID-19^23–26^. These cytokines are also associated with activation of STAT3 and PAI-1, leading to hypercoagulation in COVID-19^27–29^. Our findings corroborate these clinical observations and demonsrate an important role for endothelial ACE2 in these inflammatory responses. It is important to note that our studies were performed with the initial strain of SARS-CoV-2. Since then, the virus has undergone multiple mutations leading to different strains. However, all strains use ACE2 as the receptor for cell entry and likely induce varying inflammatory responses in host cells.

There are many possible mechanisms whereby ACE2 could influence SARS-CoV-2 inflammatory actions: (1) ACE2 as the viral entry receptor facilitates infection^4, 6^; (2) loss of enzymatic activity leads to a shift to a cardiovascular pro-damaging axis of the RAAS involving higher levels of Ang II followed by reduced production of Ang(1–7)^30, 31^; and/or (3) ACE2 regulates cell signalling directly. The role of ACE2 as a non-catalytic signalling protein is further supported in renal and intestinal epithelial cells, where ACE2 is important to the regulation of amino acid transport. In the intestine, ACE2 regulates membrane localization and function of the B(0)AT1 amino acid transporter^12^. Our findings propose another paradigm where ACE2 acts as a receptor-like protein, since pharmacological inhibition, as well as downregulation of ACE2 inhibited SARS-CoV-2 spike-induced inflammation, without any changes in ACE2 activity after exposure of human endothelial cells to the recombinant spike protein. ACE2 has been linked to inflammatory processes in other pathologies including atherosclerosis and heart disease^32–35^. Further support for ACE2 functioning as a receptor-like protein that induces pro-inflammatory signaling in the context of SARS-CoV-2, relates to our co-immunoprecipitation proteomics studies, which demonstrated that ACE2 interacts with multiple pro-inflammatory signaling molecules in rSP1-treated endothelial cells. The exact chaperone proteins of the ACE2 interactome, and specific phosphorylation sites, still await confirmation. Moreover, the fact that SARS-CoV-2 was not able to propagate in human microvascular endothelial cells supports the concept of ACE2 playing a plasma membrane receptor-like role in rS1p-induced endothelial cell damage, and that rSP1-induced inflammation is independent of virus replication in endothelial cells.

Although MLN-4760 decreased ACE2 activity in hMEC, this may not be the mechanism whereby the ACE2 inhibitor blocks rS1p injurious effects. Both, rS1p and MLN-4760 compete for the same ligand-binding domain on ACE2 structure^36^, meaning that pharmacological inhibition of ACE2 blocked rS1p activation of ACE2-dependent pathways. Interestingly, ACE2 may not be involved in all processes induced by the spike protein. Our observation that MCP-1 levels were not regulated by either ACE2 inhibition or downregulation, in addition to the differential effect observed on IL-6 production, suggests that other SARS-CoV-2 receptors in endothelial cells may be involved^37, 38^. Glucose-regulated protein 78 (GRP78), also known as the endoplasmic reticulum stress regulator BIP, has been described as another receptor for SARS-CoV-2 spike protein^39^. Under cell stress, GRP78 can translocate from the ER to the cell membrane and participate in virus entry. The spike protein-induced endothelial cell stress could cause GRP78 overexpression and translocation, leading to cytokine production, whereby it acts as a danger associated molecular patter (DAMP) activating toll-like receptors^40^. In addition, TLR activation itself may be an additional mechanism of spike protein-induced inflammation.

In conclusion, our study describes ACE2 as a pivotal signalling protein in SARS-CoV-2 spike protein-mediated activation of pro-inflammatory signalling and endothelial cell inflammation. We demonstrate that rSP1 induces endothelial inflammation via ACE2 through processes that are independent of ACE2 enzymatic activity and viral replication. Our findings define a novel role for ACE2 in SARS-CoV-2-associated endotheliitis, which may be important in cardiovascular complications associated with COVID-19.

## Methods

### Cell culture and stimulation protocols

Human dermal microvascular (hMEC) (C-12210), lymphatic (hLEC) (C-12217), aortic (hAEC) (C-12521) and pulmonary artery (hPEC) (C-12241) endothelial cells (EC) were obtained from Promocell. hMECs, hLECs and hAECs were maintained in endothelial cell growth medium MV2 containing endothelial cell growth supplement (C-22121) with penicillin/streptomycin (500 U/mL), while hAECs were maintained in endothelial cell growth medium containing endothelial cell growth supplement (C-22120) with penicillin/streptomycin (500 U/mL). Cells were rendered quiescent by maintenance in DMEM containing 0.5% fetal bovine serum (FBS) for 2 h. Cells were used from passage 5 to 9, split at a ratio of 1:3 and plated in 60 mm cell culture plates at a density of 0.8 × 106 cells. Our experimental protocol consisted in of stimulation of human ECs with recombinant SARS-CoV-2 spike protein, S1 subunit (rS1p) (accession number: QHD43416; expressed region: Val16-Gln690; catalogue number: 230-01101-500; lot number: #09G2122L; concentration: 0.75 mg/mL; RayBiotech) for short term (10 and 30 min) and long term (5 h and 24 h). All experiments were performed at 37 °C in 5% CO_2_. In some protocols using hMECs, cells were pre-incubated with MLN-4760 (440 pM) (5306160001, MilliporeSigma) or diminazine aceturate (DIZE; 190 pM) (D7770, MilliporeSigma) for 30 min or ACE2 siRNA prior to stimulations with rS1p. The final concentration of rS1p (0.66 μg/mL) used in this study was determined after a concentration curve (0.165, 0.33, 0.66 and 1.32 μg/mL) to rS1p assessing the mRNA expression of pro-inflammatory markers in hMECs (Supplemental Fig. 1).

### Real-time PCR

Total RNA was isolated using the QIAzol Lysis Reagent (Qiagen) according to the manufacturer’s instructions and diluted in nuclease-free water (Ambion/Life Technologies). cDNA was generated from total RNA using the High-Capacity cDNA Reverse Transcription Kits (Applied Biosystems). Real-time polymerase chain reaction was performed with the Applied Biosystems 7900HT Fast Real-Time PCR system, using Fast SyBr Green Master Mix (Applied Biosystems) and specific human primers to GAPDH, IL-6, MCP-1, TNFα, VCAM-1, PAI-1, thrombin, angiopoietin-2, TGFβ, preproET-1, SOD-1, catalase, glutathione peroxidase, peroxiredoxin, heme oxygenase-1 and thioredoxin; all acquired from Eurofins genomics (Glasgow, UK). Relative gene expression was calculated by the 2-∆∆Ct cycle threshold method as previously described^41^.

### ELISA to measure production of pro-inflammatory mediators

Production of MCP-1 and IL-6 was analysed in hMEC, hLEC, hAEC and hPEC supernatant after rS1p stimulation for 24 h by ELISA: human CCL2/MCP-1 DuoSet ELISA and human IL-6 DuoSet ELISA (R&D Systems). Angiopoietin-2 and ET-1 were analysed in hMEC supernatant after rS1p stimulation for 24 h using a multiplex system (HCVD2MAG-67K-02, Merck Life Science UK). Signal acquisitions were performed using the Luminex^®^ 200 system (Thermo Fisher).

### Isolation and quantification of endothelial microparticles

Medium was collected from hMEC, hLEC, hAEC and hPEC, and centrifuged for 20 min at 1500*g* to remove cell debris. 10 mL of sterile/filtered phosphatase buffer saline (PBS) was added to the media of each sample placed in ultracentrifugation tubes. Samples were centrifuged for 60 min at 20,000*g* at 4 °C (OptimaTM L-80 XP Ultracentrifuge Beckman Coulter). The microparticle (MPs) pellet was resuspended in sterile PBS for cell stimulation (~ 200 µL). MPs counting was verified using the Nanosight technology (Nanosight^®^ LM14).

### Assessing endothelial cell function using the xCELLigence assay

Cell function (hMEC) was assessed by changing of electrical impedance measured by the xCELLigence system, using the RTCA SP instrument (Roche Applied Science) as previously described^42^. Briefly, cells at a density of 2 × 10^4^ per well were plated in a 96 well E-plate containing gold microelectrode sensors on the bottom to measure cellular impedance inside each well. Cells were allowed to adhere overnight and after were treated with rS1p for 60 min. Values are represented by cell index numbers, which are a dimensionless unit of measurement representing the measurement of zero impedance when cells are absent and increasing as cells adhere, spread and divide, and analysed using RTCA software (Roche). The slope was measured based on the normalised cell index from the point in which rS1p treatment was administered.

### Measurement of reactive oxygen species (ROS)

Lucigenin-enhanced chemiluminescence was used to detect NADPH-dependent ROS generation in hMEC, hLEC, hAEC and hPEC as we previously described^43^. Briefly cells were homogenized in lysis buffer and incubated with lucigenin (5 µmol/L, Sigma-Aldrich) and NADPH (0.1 mmol/L; Sigma-Aldrich). Luminescence was measured for 29 cycles of 1.8 s each by a luminometer (Lumistar Galaxy; BMG Labtechnologies). Basal readings were recorded prior to the addition of NADPH as the substrate and were subtracted from the NADPH-dependent luminescence signal. ROS production was corrected by protein concentration and expressed as a percentage of control. Hydrogen peroxide (H_2_O_2_) levels in human endothelial cells were assessed using the Amplex Red Hydrogen Peroxide/Peroxidase Assay Kit (Molecular Probes, Life Technologies) according to manufacturer´s instructions. H_2_O_2_ levels were calculated based on standard H_2_O_2_ curves and normalised to the protein concentration for each sample. The results are expressed as percentage of control.

### ACE2 siRNA

hMECs were plated (80–90% confluent) and cultured for 24 h in growth medium (Endothelial Cell Growth Medium MV 2 supplemented with growth factors and 5% antibiotics). Cells were incubated with 20 nmol/L of ACE2 siRNA (Silencer^®^ Select, Thermo Fischer, catalog number 4392420, siRNA ID s33965) complexed with Lipofectamine™ RNAiMAX (Thermo Fischer Scientific) as transfection reagent in DMEM without serum and antibiotics for 6 h. A sequence not homologous to any gene in the vertebrate transcriptome was used as control siRNA (Silencer™ Negative Control No. 1 siRNA, Thermo Fischer, Catalog number: AM4611). After transfection, media was replaced by growth medium, and experiments were conducted 48 h after transfection.

### Immunoblotting

Total protein from hMEC was extracted in lysis buffer containing Tris (50 mmol/L, pH 8.0), NaCl (150 mmol/L), Triton X-100 (1%), SDS (0.1%), supplemented with phenylmethylsulfonyl fluoride (PMSF; 1 mmol/L), pepstatin A (1 µg/mL), leupeptin (1 µg/mL), aprotinin (1 µg/mL), sodium fluorate (10 mmol/L) and sodium orthovanadate (1 mmol/L). Total protein lysate was sonicated, cleared by centrifugation at 12,000 rpm, at 4 °C for 5 min and the pellet was discarded. Supernatants were collected and protein concentration was determined using the BCA Protein Assay kit (Thermo Fischer Scientific). Equal amounts of protein were resolved by SDS-PAGE and transferred onto a nitrocellulose membrane. Nonspecific binding sites were blocked with either non-fat dry milk or BSA, before overnight incubation in protein-specific primary antibodies to ACE2 (Proteintech, 66699-1, accession number: BC048094), ICAM-1 (Proteintech, 60299-1, accession number: BC015969), PAI-1 (Santa Cruz, 8979, accession number: P05121), phospho-ERK1/2 (Cell Signalling, 9101S, accession number: P27361, P28482), ERK1/2 (Santa Cruz, 514302, accession number: P27361, P28482), phospho-NFκB (Cell Signalling, 3033, accession number: Q04206), NFκB (Cell Signalling, 6956, accession number: Q04206), phospho-eNOS Ser1177 (Cell Signalling, 9571, accession number: P29474) and eNOS (Cell Signalling, 9572, accession number: P29474). Fluorescence-coupled antibodies (LICOR) were incubated for 1 h and were visualized by an infrared laser scanner (Odyssey Clx, LICOR). Western blotting images were quantified using the software Image Studio™ Lite. Protein expression levels were normalized to loading control β-actin (Millipore Sigma, A2228, accession number: P60709).

### Mass spectrometry protein identification and label free proteomics

hMECs were incubated with 0.66 µg/mL of rS1 protein or PBS vehicle (as control) for 24 h. CO-IP experiments were performed using Dynabeads M-270 Epoxy CO-IP kit according to the manufacturer’s recommendation, with modification in cell extraction buffer composition (1X IP buffer provided in kit, 100 mM NaCl, PMSF, 0.1% Octyl-d-glucoside). Experiments were run in triplicate (one replicate for Western Blot and duplicate for mass spectrometry identification). 2 mg of native protein lysates were prepared in modified cell extraction buffer and incubated for 1.5 h at 4 °C with slow rotation with anti-ACE2 (Abcam, ab15348, accession number: Q9BYF1) coated beads (5 µg of antibody per mg bead) (optimisation of antibody in Supplementary Fig. 11). Elution was performed in 60 µL of MS-compatible buffer provided by the manufacturer. Protein digestion was performed after reduction with dithiothreitol (56 °C) and alkylation with iodoacetamide (dark condition at RT), followed by overnight trypsin (Trypsin Gold, MS grade, Promega, USA) digestion at 37 °C. Digestion was stopped by the addition of 2 µL of formic acid and peptides were speed vacuum concentrated. Label-free quantitation of full proteome of hMECs with and without rS1 stimulation was performed with 100 µg of protein lysate. Proteins were digested after desalting (Amicon Ultra 3 kDa MWCO, Millipore), then subjected to heat denaturation (80 °C), reduction, alkylation, and trypsin digestion (1:20 ratio trypsin:protein (w/w)).

LC–MS/MS was performed on an UltiMate 3000 nano-flow system (Dionex/LC Packings) connected to an LTQ Orbitrap hybrid mass spectrometer Velos FTMS (Thermo Fisher Scientific) equipped with a nano-electrospray ion source. After loading, onto a Dionex 0.1 × 20 mm 5 µm C18 nano trap column at a flowrate of 5 µL/min, sample was eluted onto an Acclaim PepMap C18 nano column 75 µm × 50 cm, 2 µm 100 Å at a flow rate of 0.3 µL/min. The mass spectrometer was operated in data-dependent MS/MS mode scanning from 380 to 1600 amu. The top 20 multiply charged ions were selected from each full scan for MS/MS analysis using HCD at 40% collision energy. MS raw data files were searched against the Uniprot human database (UP000005640, date:22/04/2020) using label-free quantitation algorithm of MaxQuant v1.6.5.0^44^. Statistical analysis was performed using Perseus v 1.6.14.0^45^. Cytoscape v 3.8.2 with STRING app was used to generate the protein–protein interaction network^46^, followed by gene ontology enrichment by ClueGO plugin^47^ or DAVID^48^.

### SARS-CoV-2 infection

hMEC were cultured as previously described. Vero E6 (a gift from Michelle Bouloy, Institute Pasteur, France) and Vero E6 F5^50^ were maintained in Dulbecco’s Modified Eagle Medium (DMEM) supplemented with 10% foetal calf serum (FCS) at 37 °C with 5% CO_2_. hMEC cells (3.5 × 105 cells per well of a 12-well plate), and Vero E6 cells (6 × 104 cells per well of a 12-well plate) were infected with 10^5^ PFU (plaque forming units) of SARS-CoV-2 England-02 strain (GISAID accession: EPI_ISL_407073, from Public Health England) in DMEM supplemented with 2% FCS. After 2 h incubation at 37 °C the inoculum was removed, and 1 ml of DMEM supplemented with 2% FCS was added in each well. Cells were incubated at 37 °C and supernatant was harvested at 24-, 48-, and 72-h post-infection, where for each time-point a separate well was harvested. The outcome of infection was analysed by plaque assay^49^ on Vero E6 F5 cells. Briefly, cells in 12-well plates were infected with tenfold dilutions of virus samples. After 1 h incubation at 37 °C, 1 ml of overlay comprising MEM, 2% FCS, 0.6% Avicell was added per well and incubated at 37 °C for 3 days. Cell monolayers were fixed with 8% formaldehyde and plaques were visualized by staining with 0.1% Coomassie Brilliant Blue in 45% methanol and 10% glacial acetic acid. In experiments where no plaque formation was observed, a score of 5 pfu/mL was given for graphical purposes.

### HEK293-ACE2 cells generation and stimulation protocol

The ACE2-expressing HEK293 cell line (a gift from Dr. Chen Liang, Lady Davies Institute, McGill University, Canada) was generated by transduction with lentivirus particles that carry the human ACE2 gene. The lentivirus particles were produced by transfecting HEK293T cells with plasmid DNA pSPAX2 (encoding HIV-1 Gag-Pol, from Addgene, cat. 12260), VSV-G DNA (encoding VSV glycoprotein, from Addgene, cat. 8454) and ACE2 lentiviral DNA clone (encoding human ACE2 gene, from Addgene, cat. 145839). The transduced HEK293 cells were subject to selection with puromycin (2 µg/ml) (Thermo Fisher, J67236.XF), to select for cells that were stably transduced to express human ACE2. Cells were maintained in DMEM (Gibco; 11965-092) containing 10% FBS and 2 µg/ml puromycin. Cells were rendered quiescent by maintenance in DMEM containing 0.5% fetal bovine serum (FBS) for 2 h. Cells were split at a ratio of 1:3 and plated in 60 mm cell culture plates at a density of 0.8 × 106 cells. Our experimental protocol consisted in of stimulation of HEK293 and HEK293-ACE2 cells with rS1p (accession number: QHD43416; expressed region: Val16-Gln690; catalogue number: 230-01101-500; lot number: #09G2122L; concentration: 0.75 mg/mL; RayBiotech) for 24 h. All experiments were performed at 37 °C in 5% CO_2_.

### Statistical analysis

Statistical comparisons between groups were performed using two-tailed student's t-test and or one-way ANOVA. Tukey or Dunnett post-test were used as appropriate. p < 0.05 was considered statistically significant. Data analysis was conducted using GraphPad Prism^®^ 8.0 (GraphPad Software Inc.). Data are expressed as mean ± SEM.

### Supplementary Information

Supplementary Figures.

Supplementary Information.

## Data Availability

The datasets generated during and/or analysed during the current study are available from the corresponding authors on reasonable request. Correspondence and requests for materials should be addressed to A.C.M.
