# Supplementary material for: SARS-CoV-2 spike protein induces endothelial inflammation via ACE2 independently of viral replication
Source: Sci Rep. 2023 Aug 28;13:14086. doi: 10.1038/s41598-023-41115-3 (PMC10462711; doi:10.1038/s41598-023-41115-3)

SUPPLEMENTARY MATERIAL

SARS-CoV-2 spike protein induces endothelial inflammation via ACE2 independently of viral replication

Augusto C Montezano, PhD[1,2*+], Livia L Camargo, PhD[1+], Sheon Mary, PhD[2], Karla Neves, PhD[2,3], Francisco Rios, PhD[1], Ross Stein[2], Rheure A Lopes, PhD[2], Wendy Beattie, MSc[2], Jacqueline Thomson, MSc[2], Vanessa Herder, PhD[4], Agnieszka M Szemiel, PhD[4], Steven McFarlane, PhD[4], Massimo Palmarini, PhD[4], Rhian M Touyz, MD, PhD[1,2*].

1. Research Institute of the McGill University Health Centre – Montreal, Canada
2. School of Cardiovascular and Metabolic Health – University of Glasgow, UK
3. Strathclyde Institute of Pharmacy and Biomedical Sciences, University of Strathclyde, Glasgow, UK
4. MRC - University of Glasgow Centre for Virus Research, UK

+ Authors contributed equally to the study

Corresponding Authors:

Augusto C Montezano, PhD (ORCID: 0000-0002-8658-7994) and Rhian M Touyz, MD, PhD (ORCID: 0000-0003-0670-0887)

Research Institute of the McGill University Health Centre (RI-MUHC)
Site Glen - Block E - Office: E01.3362
1001, boul. Decarie, Montreal, Quebec, CANADA, H4A3J1
email: augusto.montezano@muhc.mcgill.ca; rhian.touyz@mcgill.ca
Phone: 514-9341934 ext: 37841

Supplementary Figure Legends

Supplementary Fig. S1 – rS1p concentration-dependent effects on mRNA expression of pro-inflammatory markers in hMECs. Human endothelial cells (hMEC) were exposed to increasing concentrations of rS1p (0.165, 0.33, 0.66, 1.32 µg/mL) for 5h and mRNA expression of IL-6 (A), TNFα (B), MCP-1 (C) and VCAM-1 (D) was assessed by RT-PCR (n=5). Data are expressed as ± SEM; * $p<0.05$ control (non-stimulated cells) vs. rS1p stimulated cells at different concentrations after 1-way ANOVA followed by Dunnett's post-hoc test.

Supplementary Fig. S2 – rS1p increases pro-inflammatory gene expression and short term microparticle formation in hMECs. Human endothelial cells (hMEC) were stimulated with rS1p (0.66 µg/mL) for 5h and 24h for assessment of TNFα (A), VCAM-1 (B), PAI-1 (C), thrombin (D), angiopoetin-2 (E) mRNA expression and short term microparticles formation (F) (n=6). Data are expressed as ± SEM; * $p<0.05$ control (Ctl) (non-stimulated cells) vs. rS1p stimulated cells after 1-way ANOVA followed by Tukey's post-hoc test.

Supplementary Fig. S3 – rS1p does not change TGFβ and preproET-1 gene expression in hMECs. Human endothelial cells (hMEC) were stimulated with rS1p (0.66 µg/mL) for 5h and 24h for assessment of TGFβ (A) and preproET-1 (B) mRNA expression (n=5). Data are expressed as ± SEM.

Supplementary Fig. S4 – rS1p does not induce ROS production in hMECs. Human endothelial cells (hMEC) were stimulated with rS1p (0.66 µg/mL) for 10 and 30 minutes for assessment of ROS (A) and H_2O_2 production (B) (n=5-6). Data are expressed as ± SEM.

Supplementary Fig. S5 – rS1p does not change gene expression of antioxidants in hMECs. Human endothelial cells (hMEC) were stimulated with rS1p (0.66 µg/mL) for 5h and 24h for assessment of SOD1 (A), catalase (B), glutathione peroxidase (GPX) (C), peroxiredoxin (PRDX) (D), heme oxygenase-1 (HO-1) (E) and thioredoxin (F) mRNA expression (n=6). Data are expressed as ± SEM.

Supplementary Fig. S6 – rS1p increases gene expression of pro-inflammatory mediators and induces microparticle formation in hLECs. Human endothelial cells (hLEC) were stimulated with rS1p (0.66 µg/mL) for 5h and 24h for assessment of IL-6 (A) and MCP-1 (B) mRNA expression; IL-6 (C) and MCP-1 (D) production; microparticles formation at long (E) and short term (F); and ROS (G) and H2O2 (H) production (n=5-6). Data are expressed as ± SEM; * $p<0.05$ control (Ctl) (non-stimulated cells) vs. rS1p stimulated cells after student's t-test (C, D, E) or 1-way ANOVA followed by Tukey's post-hoc test.

Supplementary Fig. S7 – rS1p increases pro-inflammatory markers gene expression and microparticle formation in hAECs. Human endothelial cells (hAEC) were stimulated with rS1p (0.66 µg/mL) for 5h and 24h for assessment of IL-6 (A) and MCP-1 (B) mRNA expression; IL-6 (C) and MCP-1 (D) production; microparticles formation at long (E) and short term (F); and ROS (G) and H2O2 (H) production (n=5-6). Data are expressed as ± SEM; * $p<0.05$ control (Ctl) (non-stimulated cells) vs. rS1p stimulated cells after student's t-test (C, D, E) or 1-way ANOVA followed by Tukey's post-hoc test.

Supplementary Fig. S8 – rS1p increases pro-inflammatory markers gene expression and microparticle formation in hPECs. Human endothelial cells (hPEC) were stimulated with rS1p (0.66 µg/mL) for 5h and 24h for assessment of IL-6 (A) and MCP-1 (B) mRNA expression; IL-6 (C) and MCP-1 (D) production; microparticles formation at long (E) and short term (F); and ROS (G) and H2O2 (H) production (n=5-6). Data are expressed as ± SEM; * $p<0.05$ control (Ctl) (non-stimulated cells) vs. rS1p stimulated cells after student's t-test (C, D, E) or 1-way ANOVA followed by Tukey's post-hoc test.

Supplementary Fig. S9 – rS1p effects in pro-inflammatory markers mRNA expression after treatment with the ACE2 inhibitor MLN-4760 in human endothelial cells. Human endothelial cells (hMEC) were stimulated with rS1p (0.66 µg/mL) for 5h for assessment of IL-6 (A), MCP-1 (B), PAI-1 (C) and VCAM-1 (D) mRNA expression in the presence or absence of MLN-4760 (440 pmol), an ACE2

inhibitor (n=10-11). Data are expressed as ± SEM; * $p<0.05$ control (Ctl) (non-stimulated cells) vs. rS1p stimulated cells; † $p<0.05$ rS1p stimulated cells vs. rS1p stimulated cells treated with MLN-4760 after 1-way ANOVA followed by Tukey's post-hoc test.

Supplementary Fig. S10 – rS1p effects in pro-inflammatory markers mRNA expression after treatment with the ACE2 activator DIZE in human endothelial cells. Human endothelial cells (hMEC) were stimulated with rS1p (0.66 μg/mL) for 5h for assessment of IL-6 (A), MCP-1 (B) and TNFα (C) mRNA expression in the presence or absence of DIZE (190 pmol), an ACE2 activator (n=10). Data are expressed as ± SEM; * $p<0.05$ control (Ctl) (non-stimulated cells) vs. rS1p stimulated cells in the presence or absence of DIZE after 1-way ANOVA followed by Tukey's post-hoc test.

Supplementary Fig. S11 – characterization of ACE2 co-immunoprecipitation coupled mass spectrometry protein identification and label free proteomics. Optimisation of anti-ACE2 antibody for co-immunoprecipitation (A). Antibodies were used from Abcam (ab15348) and proteintech (66699-1). Lane labelling: M - protein ladder, L - protein lysate load, U - unbound fraction, 1 to 3 - washes, E - elute. Western blot with primary anti-ACE2 (Abcam), secondary anti-rabbit Alexa800. Native/non-denaturing protein isolation was done to maintain the protein-protein interaction. This led to identification of more soluble ACE2 fractions (bands below 70kDa) along with glycosylated and high molecular weight ACE2 (bands above 100kDa). Co-immunoprecipitation of Anti-ACE2 (Abcam) (B). Lane labelling: M - protein ladder, 1 - protein lysate load, 2 - unbound fraction, 3 to 6 - washes, 7- elute, 8 - elution from beads with anti-ACE2 (negative control). Final protein load amount was of 10% of each fraction (except elution fraction). 4-12% Bis tris gel, nitrocellulose membrane with 5% BSA was used. ClueGO enriched gene ontology term for biological processes (C). Each circle represents a group of gene (identified in ACE2 Co-IP) that belong to either a particular biological function. Size of the circle represents the enrichment significance (cut off Bonferroni FDR <0.5, at least 3 gene per group)

Supplementary Fig. S12 – rS1p or ACE2 siRNA does not alter ET-1 and angiopoetin-2 levels in human endothelial cells. Human endothelial cells (hMEC) were stimulated with rS1p (0.66 μg/mL) for 24h for assessment of ET-1 (A) and angiopoetin-2 (B) production in the presence or absence of ACE2 siRNA (n=8). Data are expressed as ± SEM.

SUPPLEMENTARY FIGURE S1

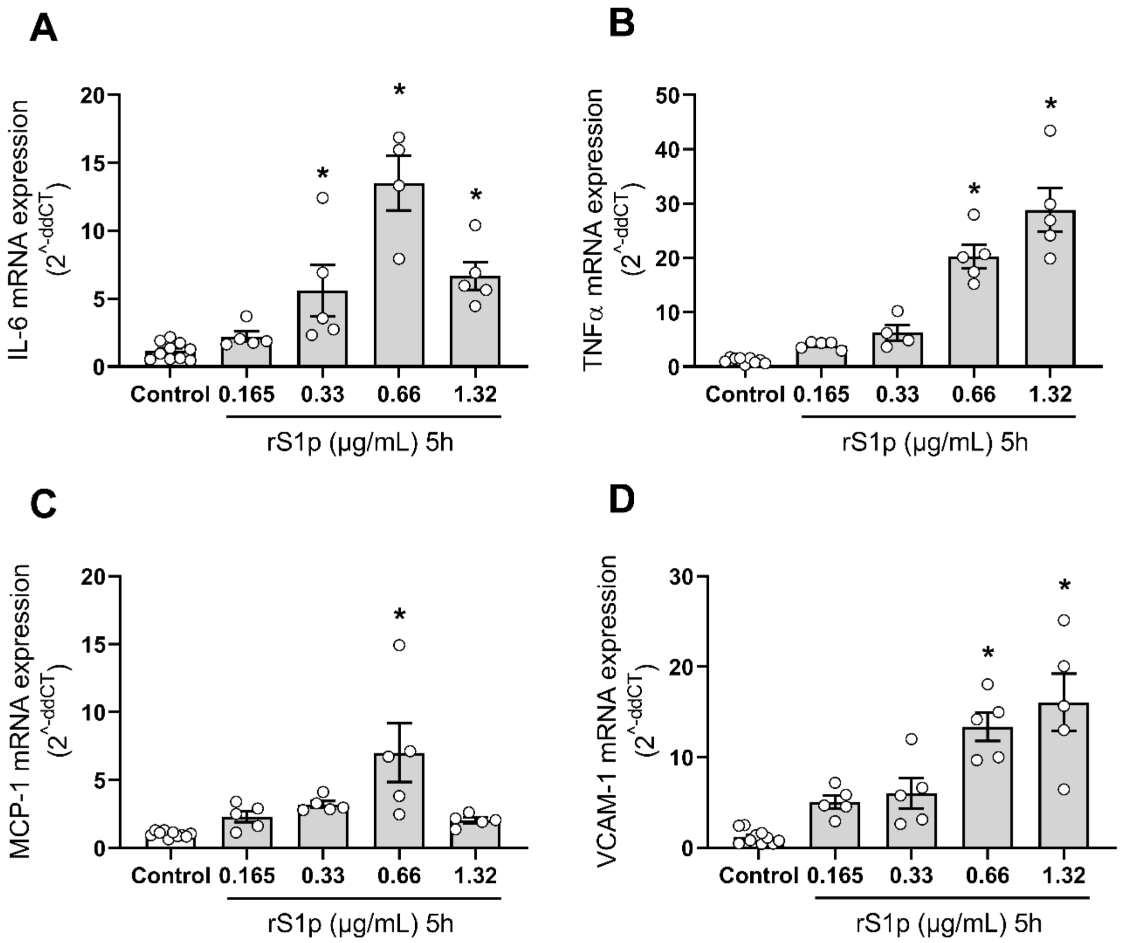

SUPPLEMENTARY FIGURE S2

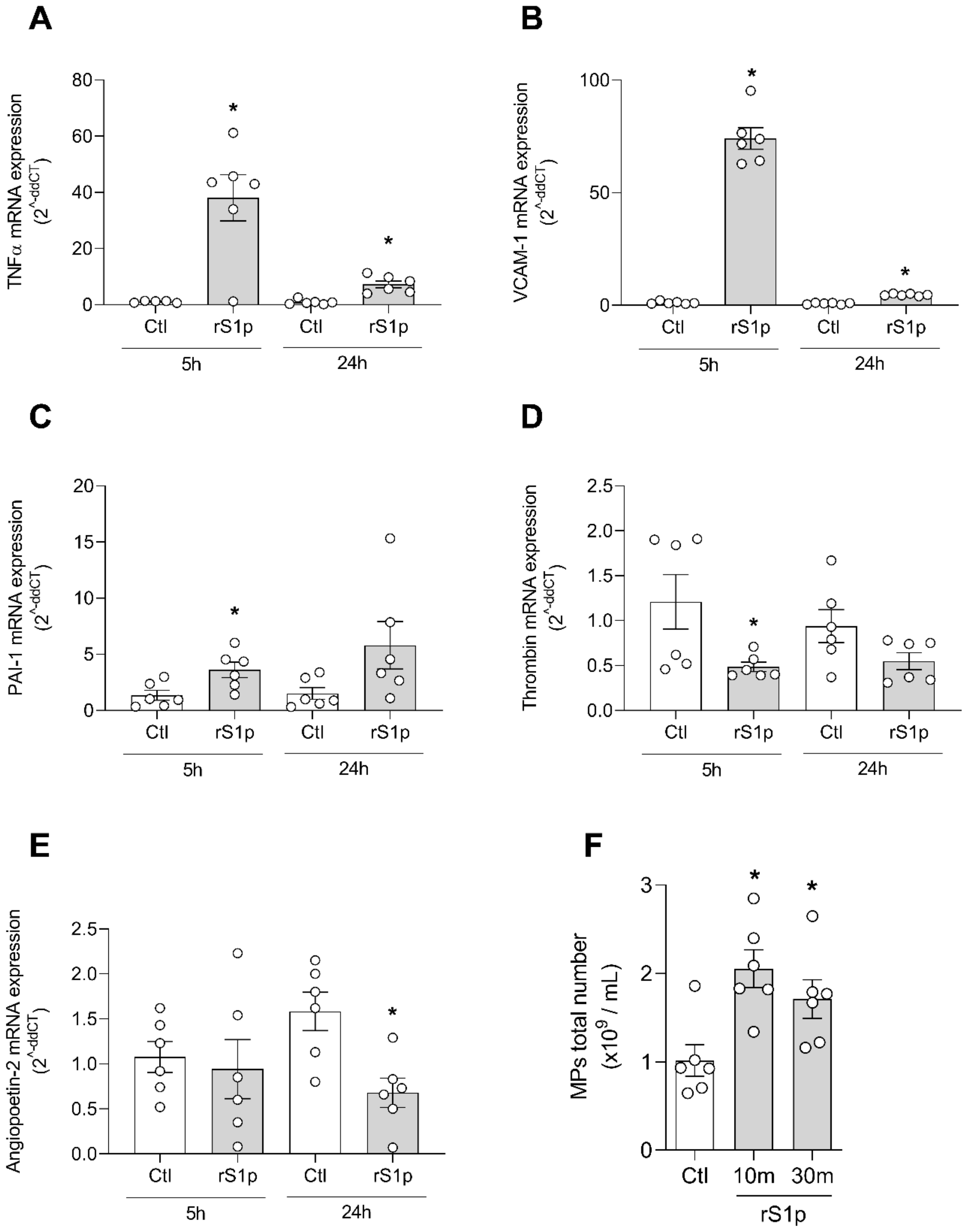

SUPPLEMENTARY FIGURE S3

A

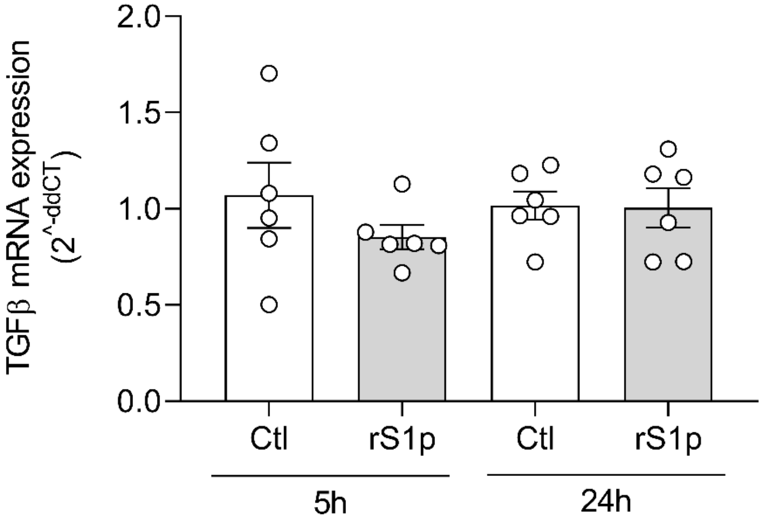

B

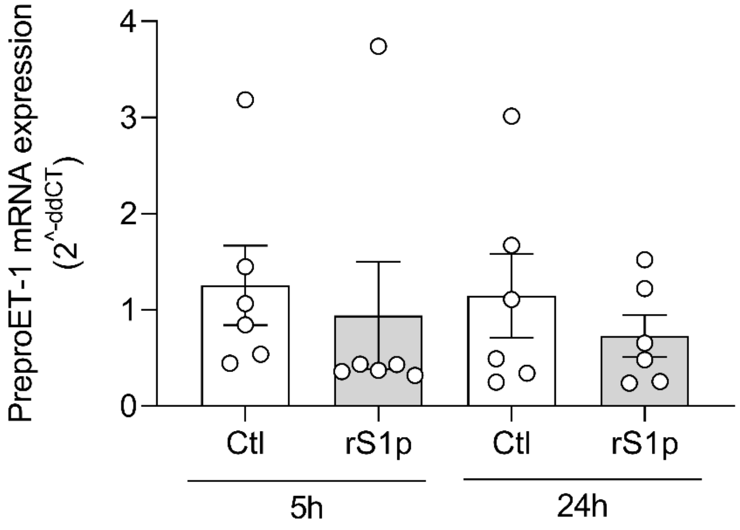

SUPPLEMENTARY FIGURE S4

A

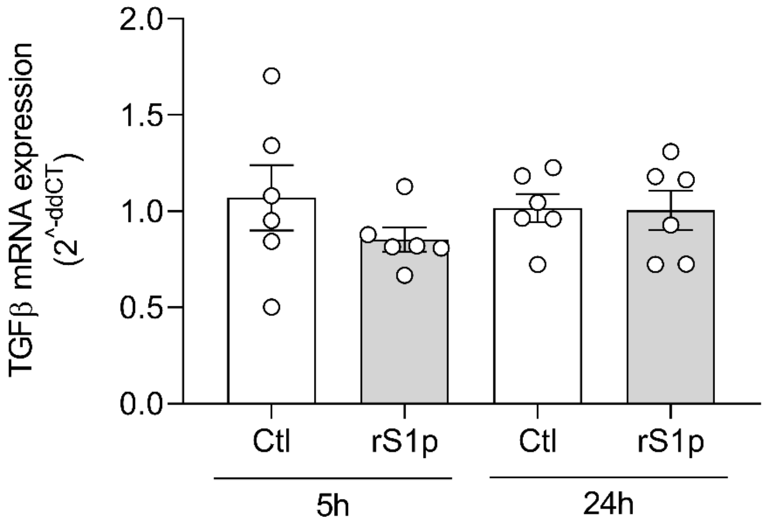

B

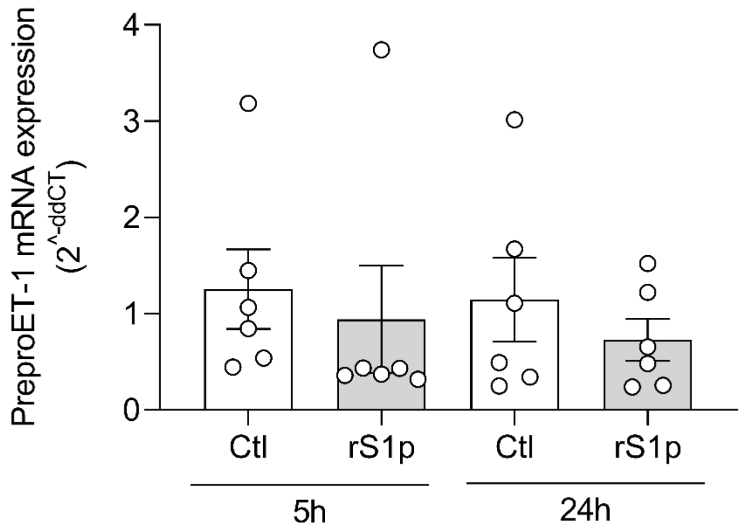

SUPPLEMENTARY FIGURE S5

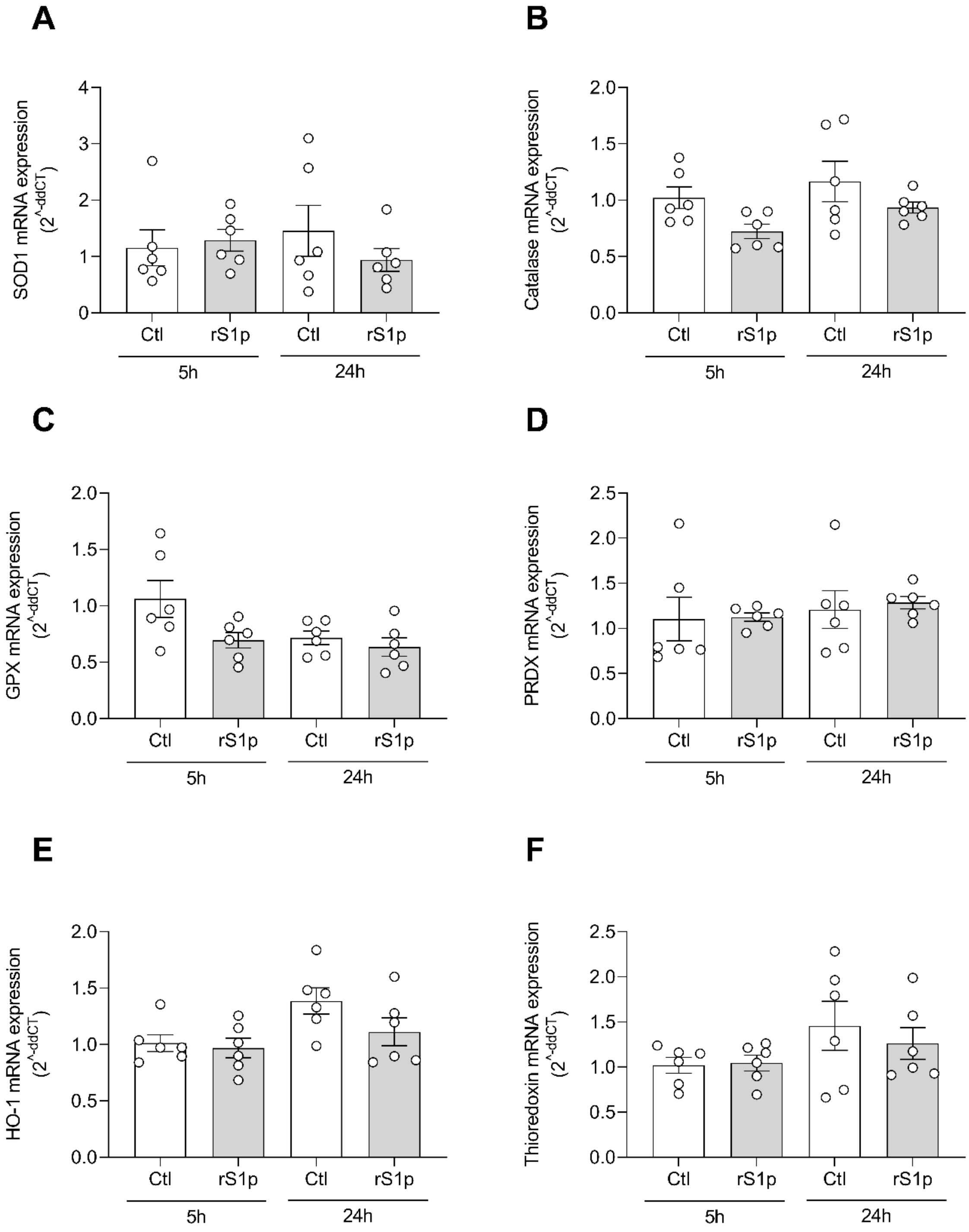

SUPPLEMENTARY FIGURE S6

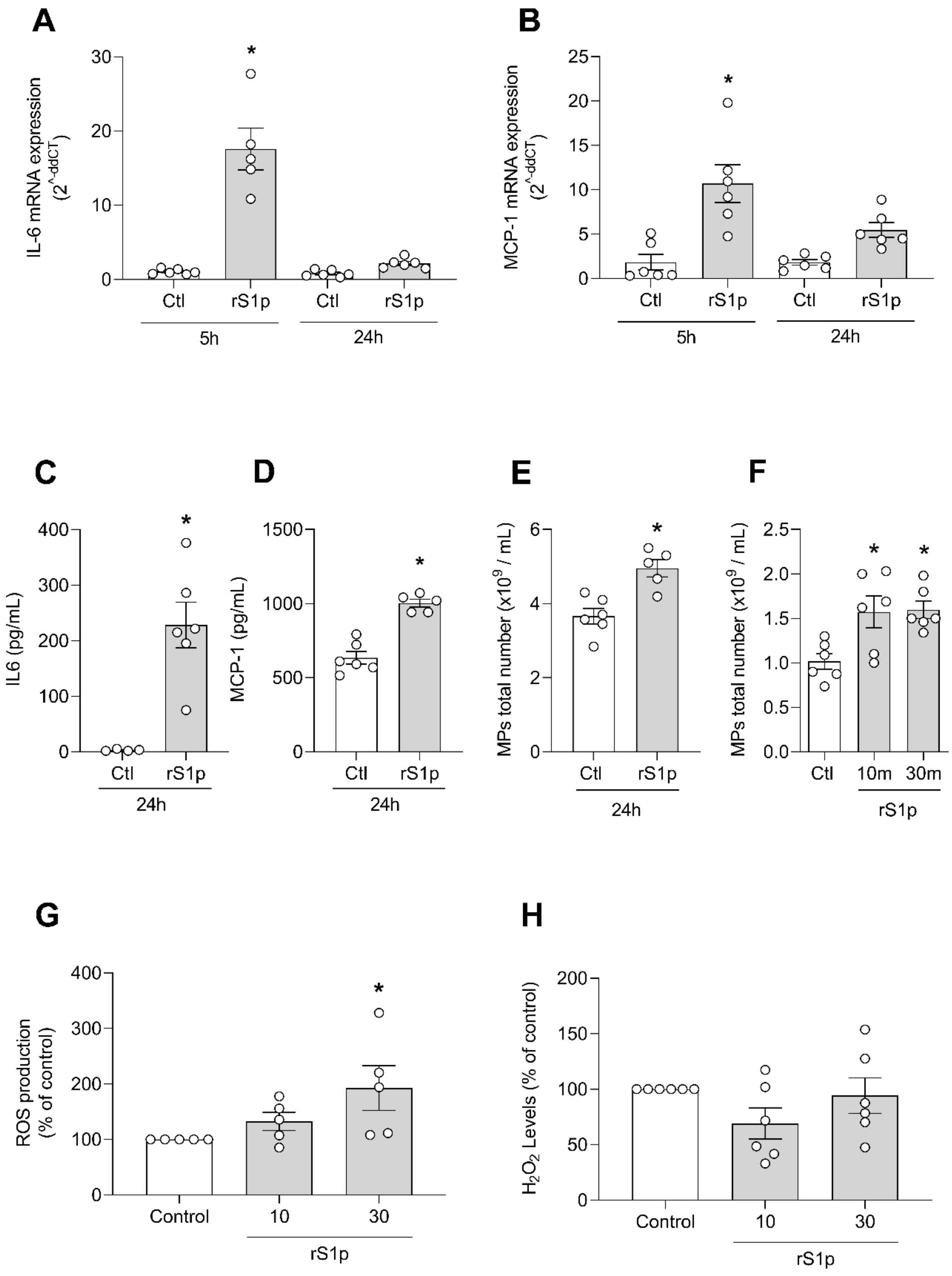

SUPPLEMENTARY FIGURE S7

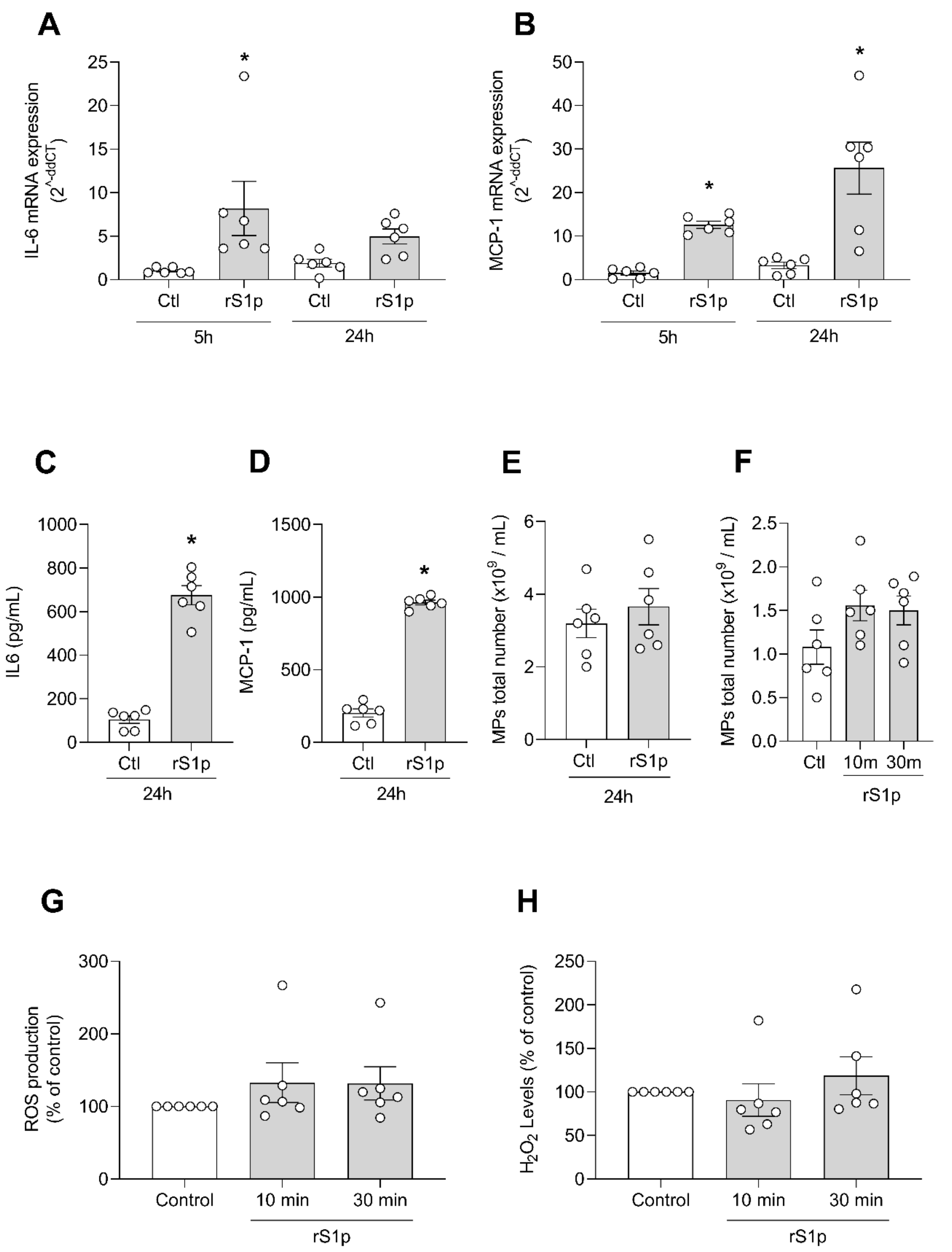

SUPPLEMENTARY FIGURE S8

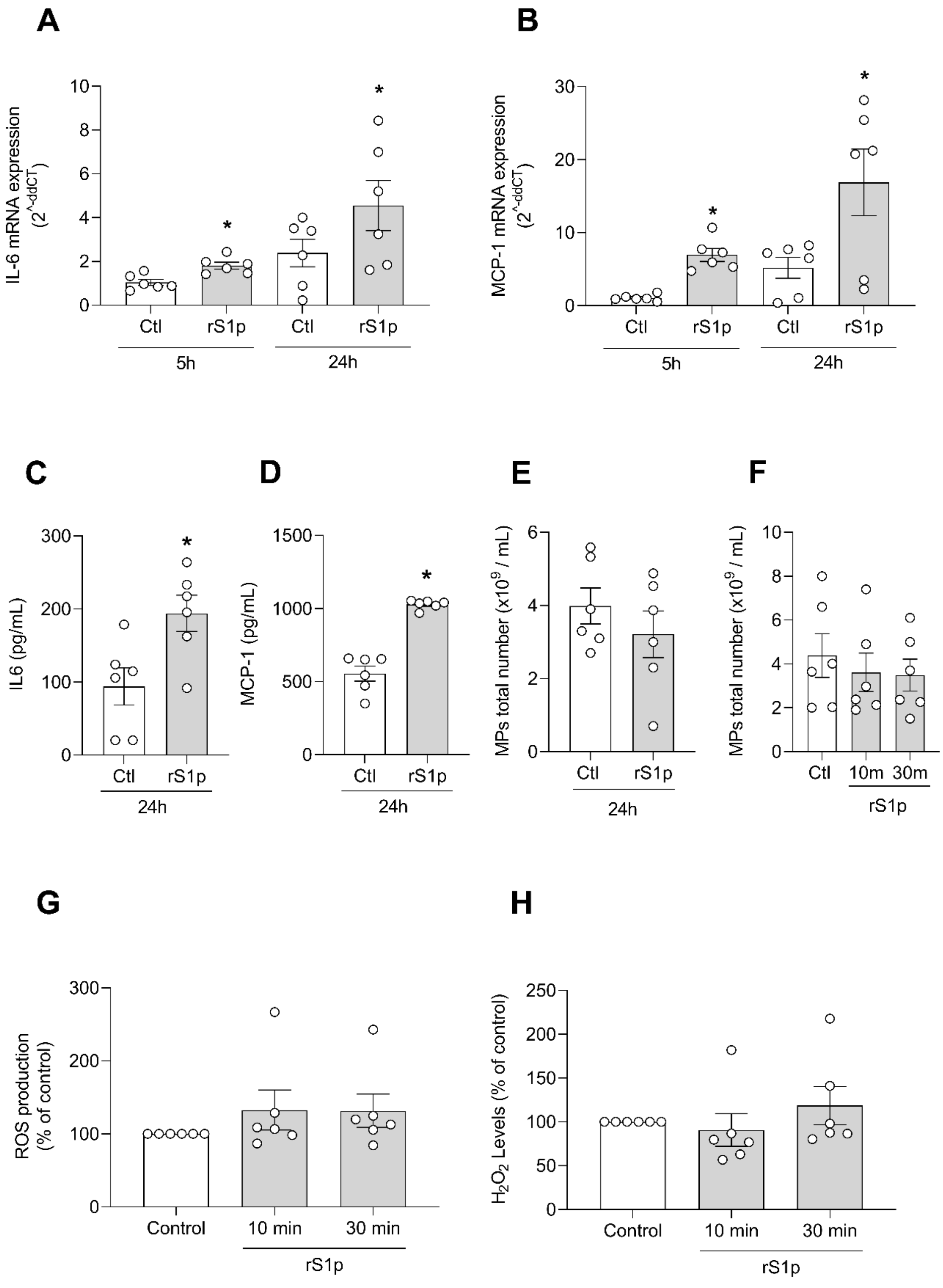

SUPPLEMENTARY FIGURE S9

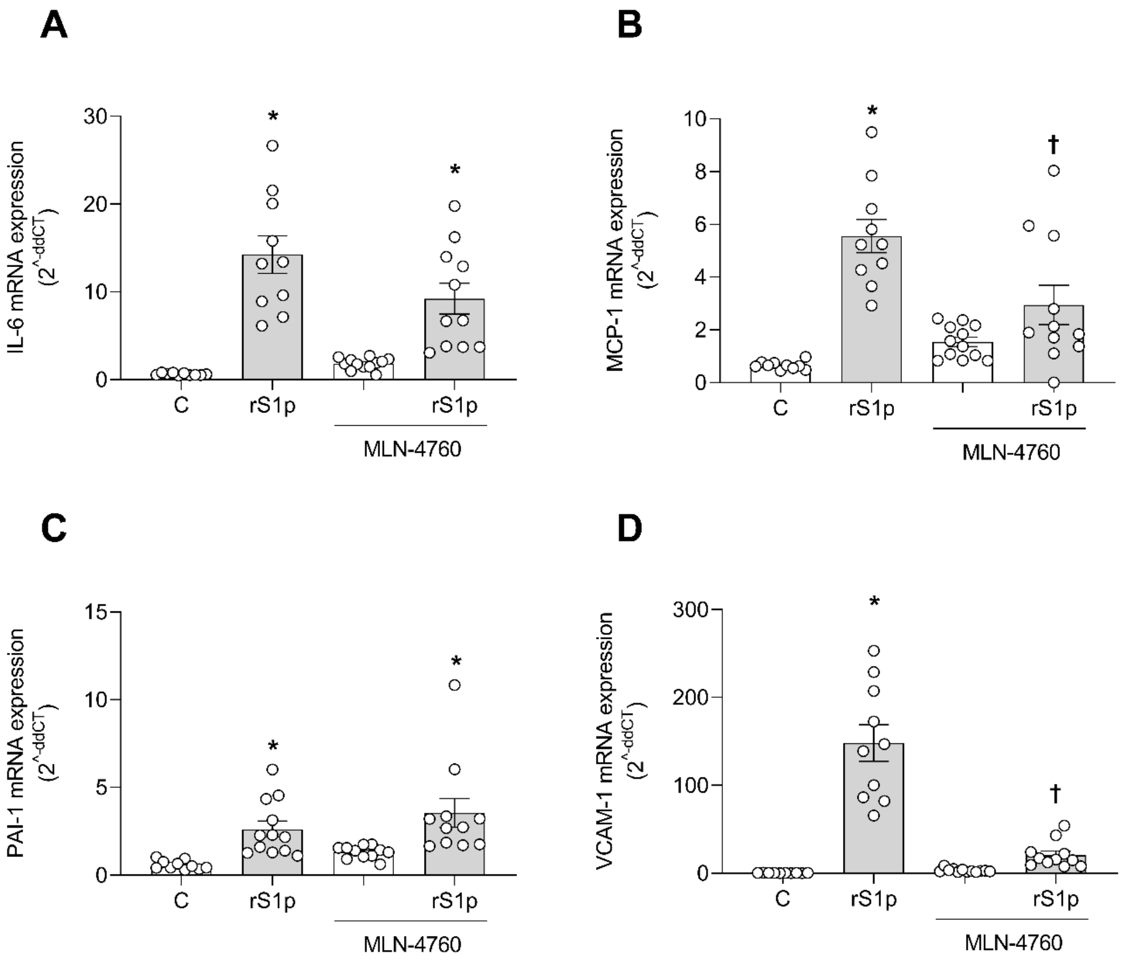

SUPPLEMENTARY FIGURE S10

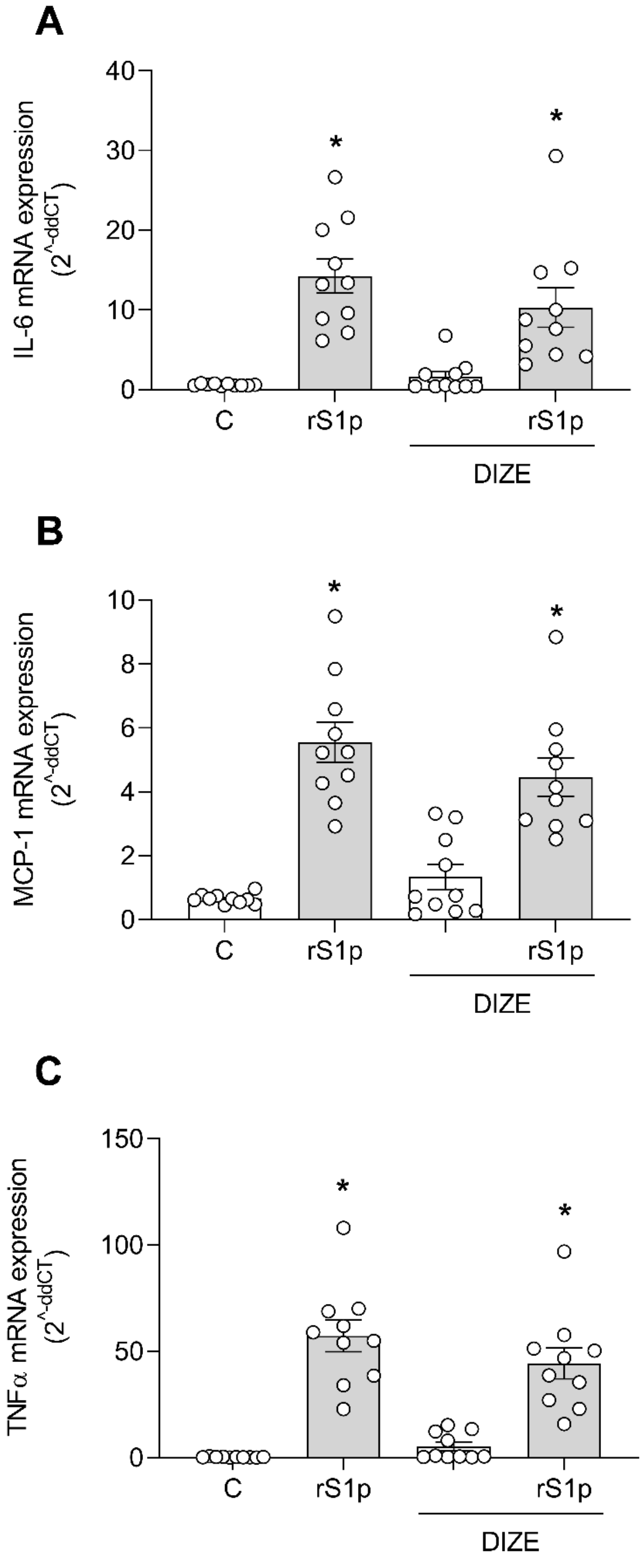

SUPPLEMENTARY FIGURE S11

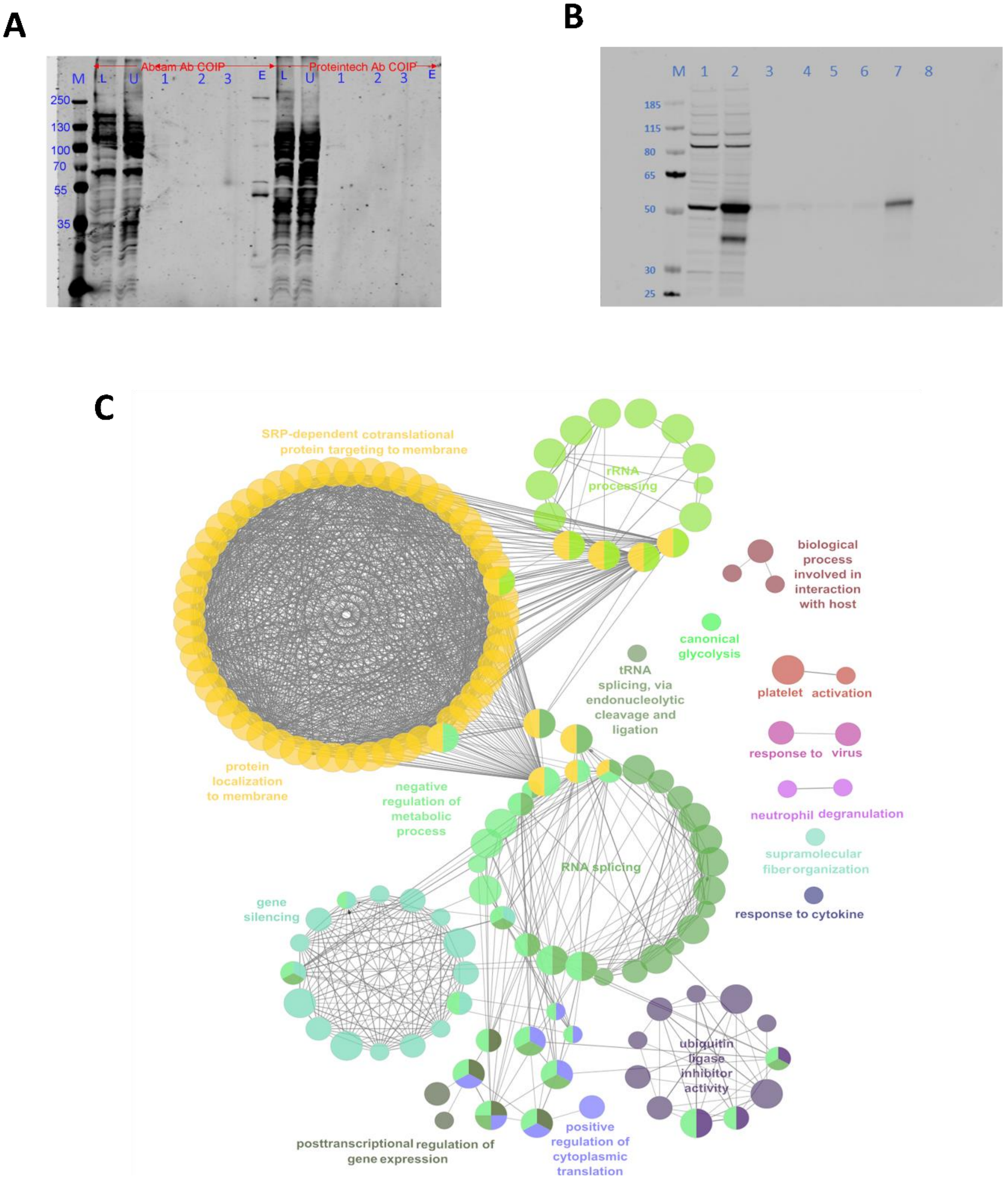

SUPPLEMENTARY FIGURE S12

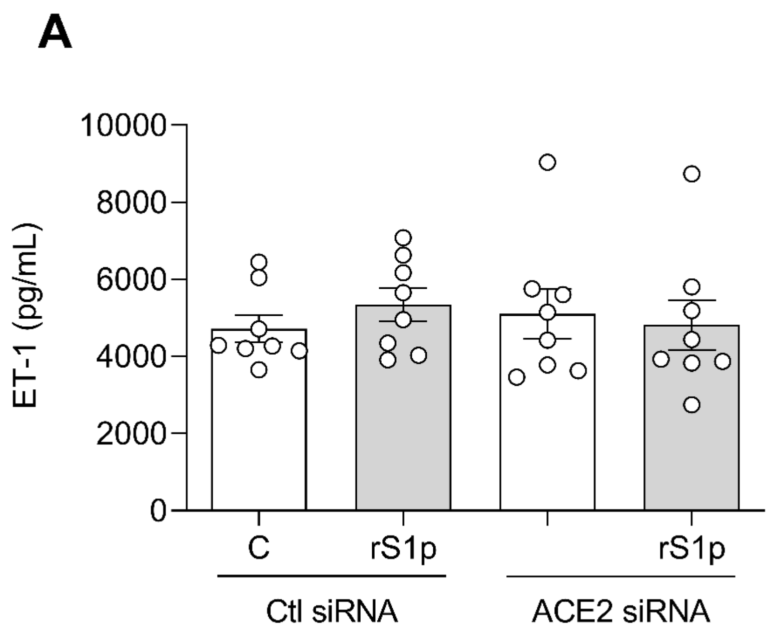

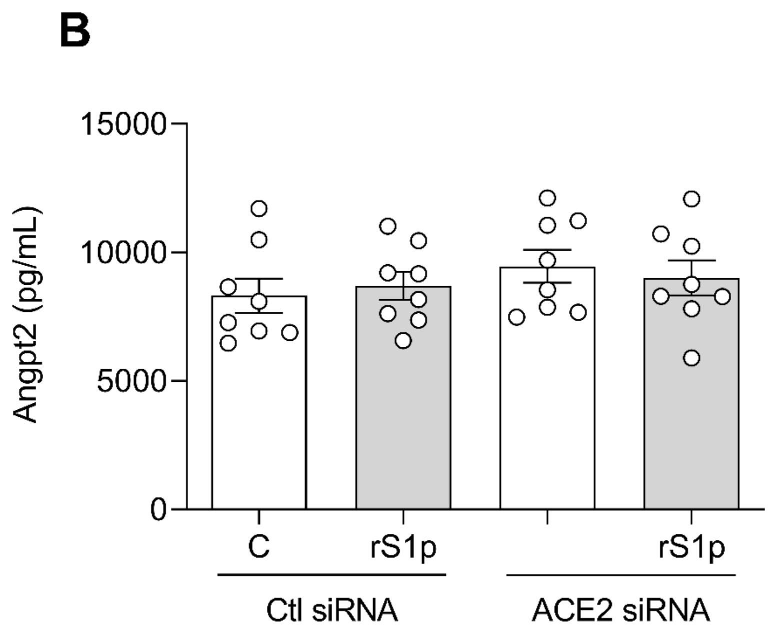

SUPPLEMENTARY FIGURE S13 - ORIGINAL BLOTS – FIGURE 3B – ACE2

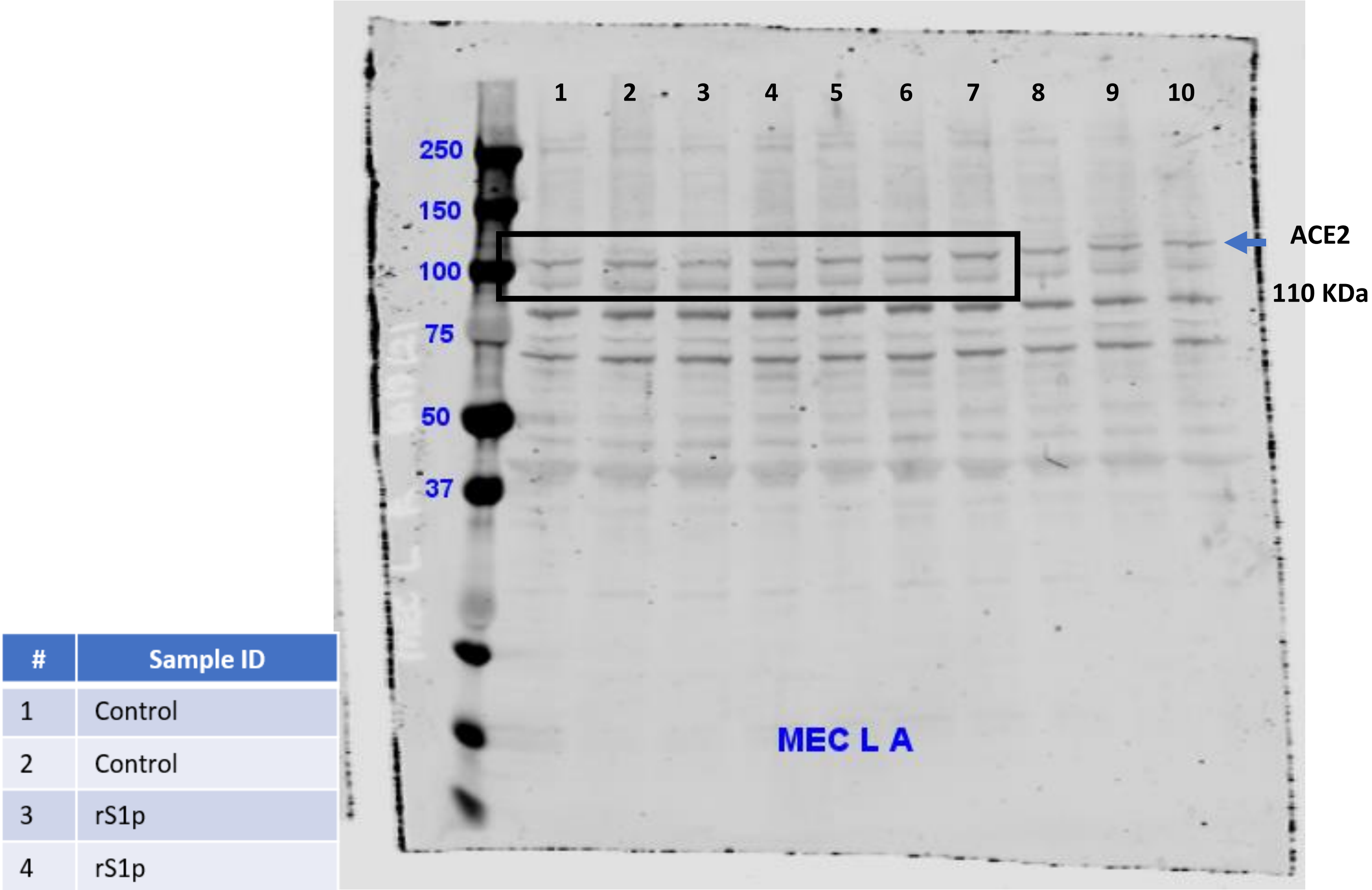

#	Sample ID
1	Control
2	Control
3	rS1p
4	rS1p
5	MLN-4760
6	rS1p + MLN-4760
7	rS1p + MLN-4760
8	DIZE
9	rS1p + DIZE
10	rS1p + DIZE

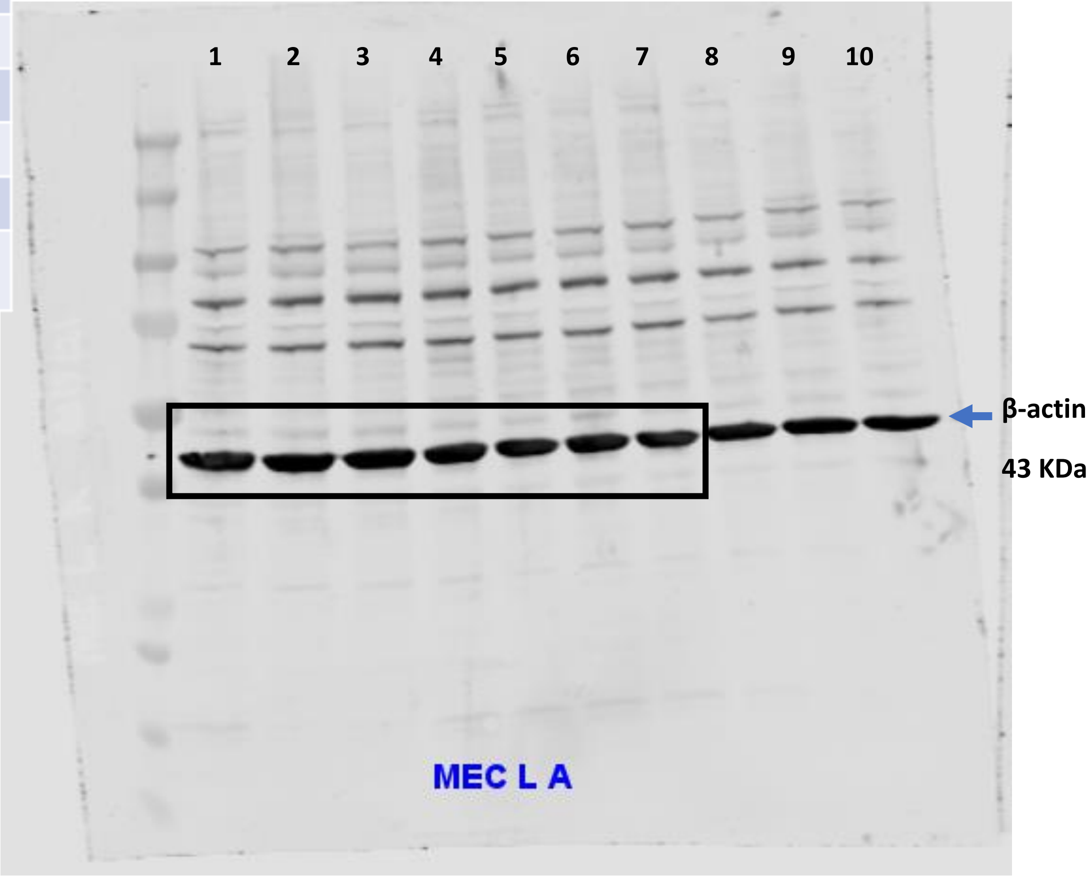

SUPPLEMENTARY FIGURE S14 – ORIGINAL BLOTS - FIG 3F/3G – ICAM-1 AND PAI-1

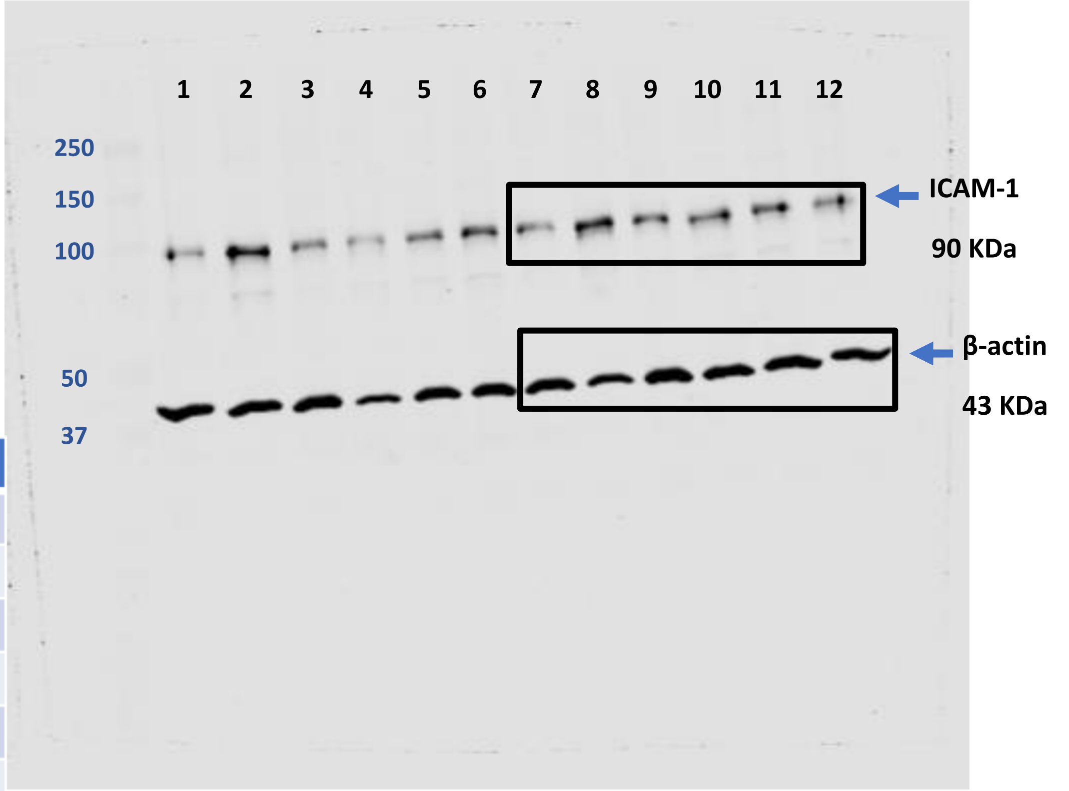

#	Sample ID
1	Control
2	rS1p
3	Control
4	rS1p
5	MLN-4760 + control
6	MLN-4760 + rS1p
7	Control
8	rS1p
9	Control
10	rS1p
11	MLN-4760 + control
12	MLN-4760 + rS1p

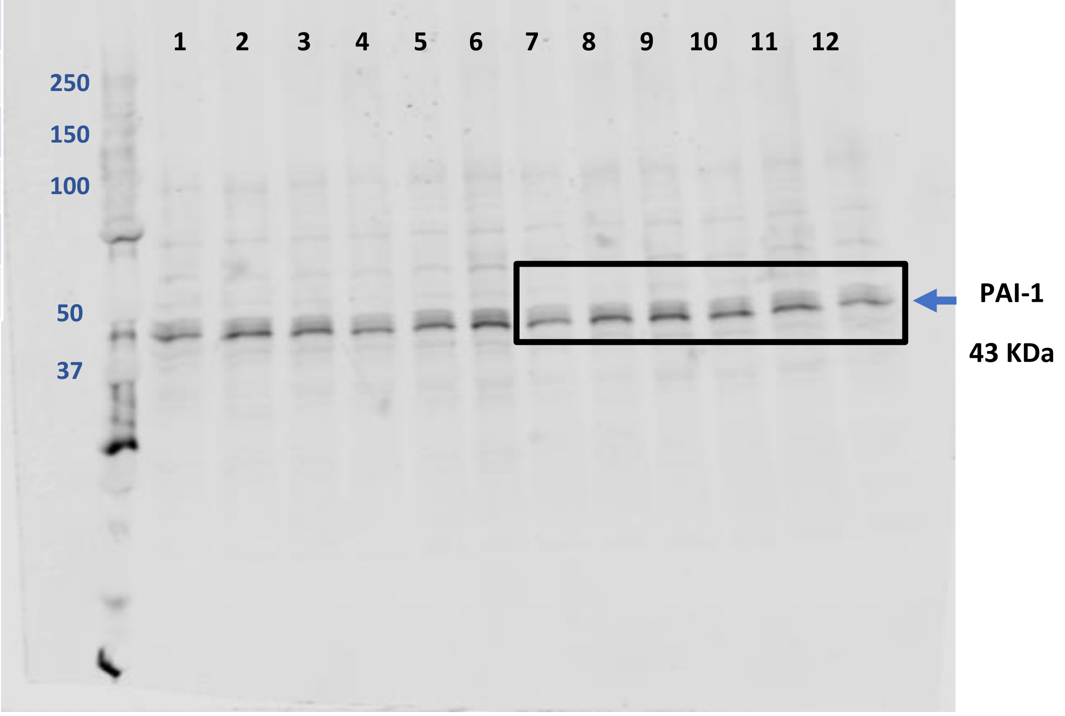

SUPPLEMENTARY FIGURE S15 - ORIGINAL BLOTS – FIGURE 4A – ACE2

1	Ctl	Ctl siRNA	
2	S1 24h		MEC1
3	Ctl	ACE2 siRNA	exp7
4	S1 24h		
5	Ctl	Ctl siRNA	
6	S1 24h		MEC2
7	Ctl	ACE2 siRNA	exp8
8	S1 24h		
9	Ctl	Ctl siRNA	
10	S1 24h		MEC3
11	Ctl	ACE2 siRNA	exp9
12	S1 24h		

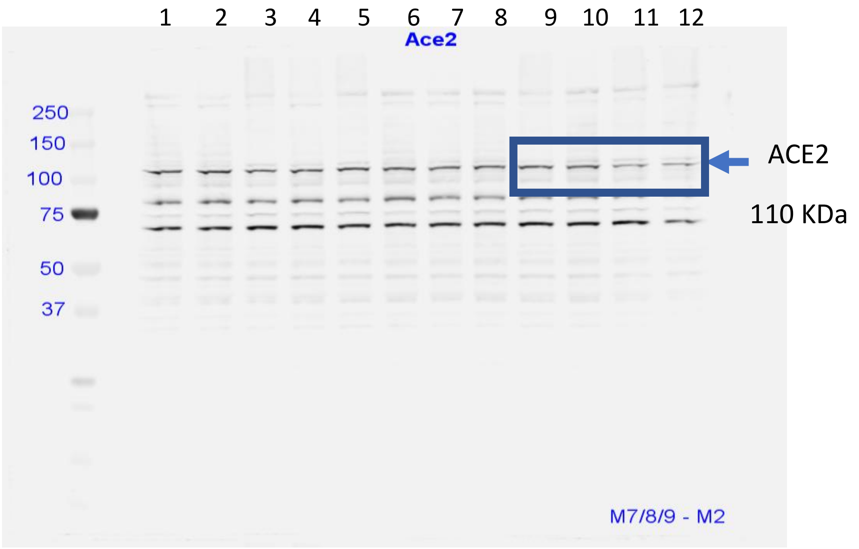

1	Ctl	Ctl siRNA	
2	S1 24h		MEC1
3	Ctl	ACE2 siRNA	exp7
4	S1 24h		
5	Ctl	Ctl siRNA	
6	S1 24h		MEC2
7	Ctl	ACE2 siRNA	exp8
8	S1 24h		
9	Ctl	Ctl siRNA	
10	S1 24h		MEC3
11	Ctl	ACE2 siRNA	exp9
12	S1 24h		

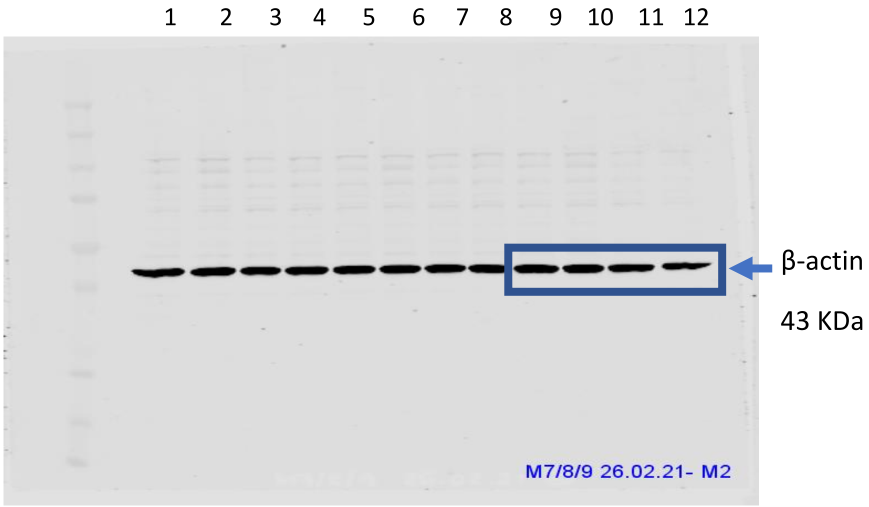

SUPPLEMENTARY FIGURE S16 - ORIGINAL BLOTS – FIGURE 4D – ICAM-1

1	Ctl	Ctl siRNA	
2	S1 24h		MEC1
3	Ctl	ACE2 siRNA	exp7
4	S1 24h		
5	Ctl	Ctl siRNA	
6	S1 24h		MEC2
7	Ctl	ACE2 siRNA	exp8
8	S1 24h		
9	Ctl	Ctl siRNA	
10	S1 24h		MEC3
11	Ctl	ACE2 siRNA	exp9
12	S1 24h		

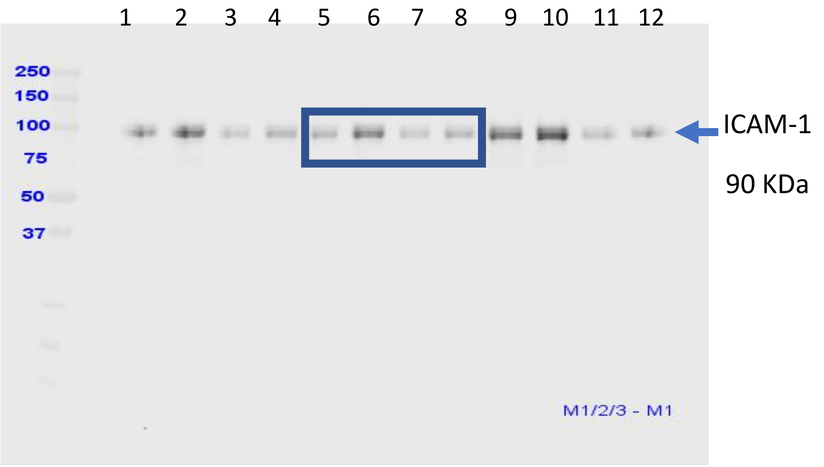

1	Ctl	Ctl siRNA	
2	S1 24h		MEC1
3	Ctl	ACE2 siRNA	exp7
4	S1 24h		
5	Ctl	Ctl siRNA	
6	S1 24h		MEC2
7	Ctl	ACE2 siRNA	exp8
8	S1 24h		
9	Ctl	Ctl siRNA	
10	S1 24h		MEC3
11	Ctl	ACE2 siRNA	exp9
12	S1 24h		

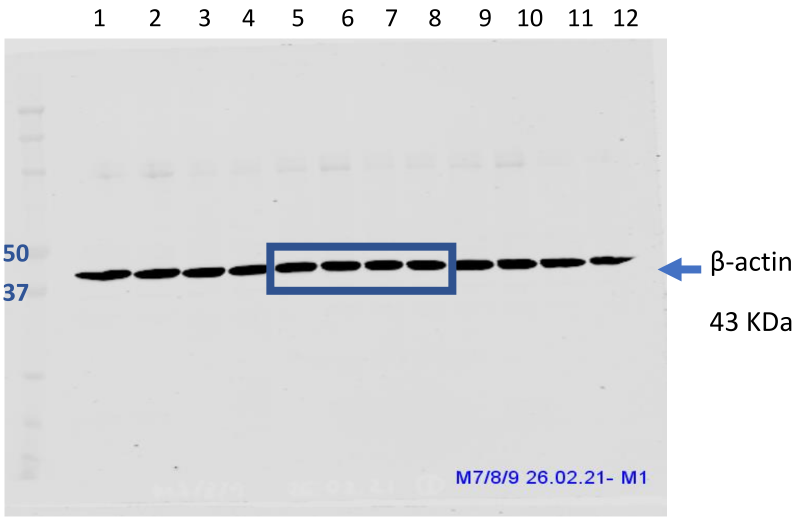

SUPPLEMENTARY FIGURE S17 - ORIGINAL BLOTS – FIGURE 4E – PAI-1

1	Ctl	Ctl siRNA	
2	S1 24h		MEC1
3	Ctl	ACE2 siRNA	exp7
4	S1 24h		
5	Ctl	Ctl siRNA	
6	S1 24h		MEC2
7	Ctl	ACE2 siRNA	exp8
8	S1 24h		
9	Ctl	Ctl siRNA	
10	S1 24h		MEC3
11	Ctl	ACE2 siRNA	exp9
12	S1 24h		

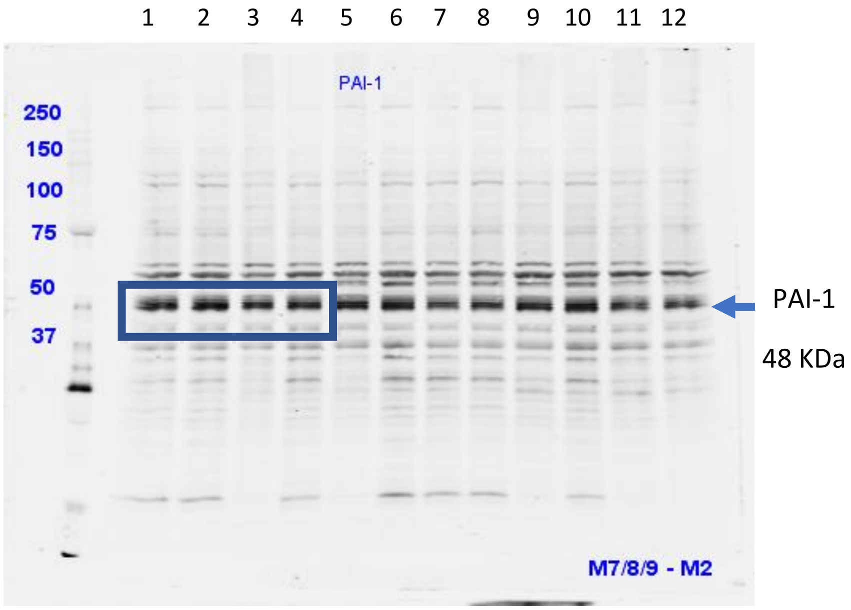

1	Ctl	Ctl siRNA	
2	S1 24h		MEC1
3	Ctl	ACE2 siRNA	exp7
4	S1 24h		
5	Ctl	Ctl siRNA	
6	S1 24h		MEC2
7	Ctl	ACE2 siRNA	exp8
8	S1 24h		
9	Ctl	Ctl siRNA	
10	S1 24h		MEC3
11	Ctl	ACE2 siRNA	exp9
12	S1 24h		

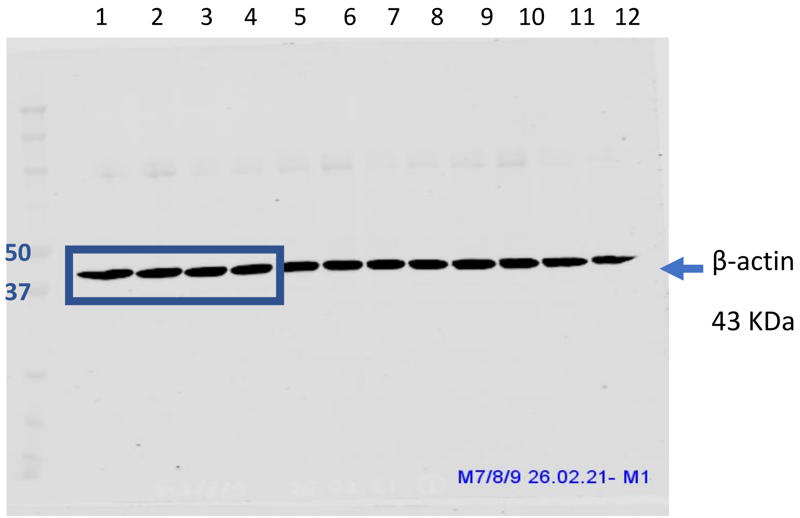

SUPPLEMENTARY FIGURE S18 - ORIGINAL BLOTS – FIGURE 5A – ERK1/2

			M2
1	Ctl	Ctl siRNA	
2	S1 24h		
3	Ctl	ACE2 siRNA	exp4
4	S1 24h		
5	Ctl	Ctl siRNA	
6	S1 24h		
7	Ctl	ACE2 siRNA	exp5
8	S1 24h		
9	Ctl	Ctl siRNA	
10	S1 24h		
11	Ctl	ACE2 siRNA	exp6
12	S1 24h		

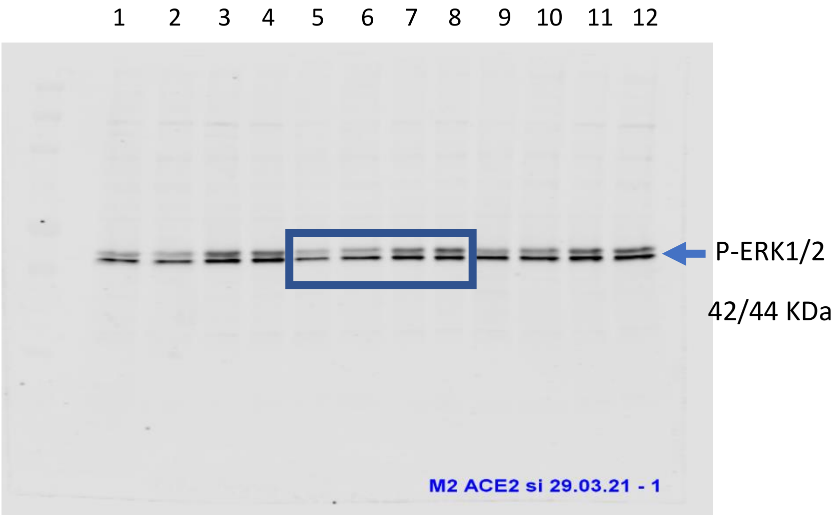

			M2
1	Ctl	Ctl siRNA	
2	S1 24h		
3	Ctl	ACE2 siRNA	exp4
4	S1 24h		
5	Ctl	Ctl siRNA	
6	S1 24h		
7	Ctl	ACE2 siRNA	exp5
8	S1 24h		
9	Ctl	Ctl siRNA	
10	S1 24h		
11	Ctl	ACE2 siRNA	exp6
12	S1 24h		

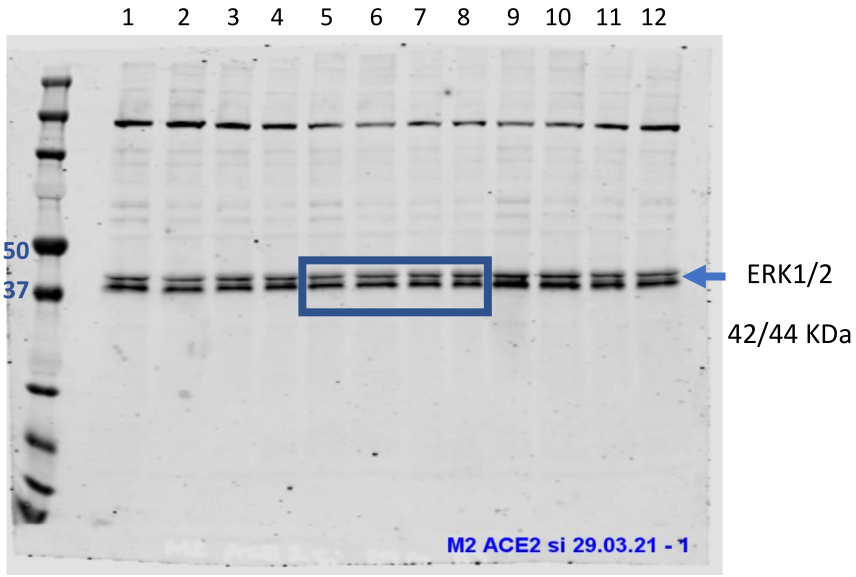

SUPPLEMENTARY FIGURE S19 - ORIGINAL BLOTS – FIGURE 5B – NFkB

			M1/2/3
1	Ctl	Ctl siRNA	
2	S1 24h		
3	Ctl	ACE2 siRNA	exp1
4	S1 24h		
5	Ctl	Ctl siRNA	
6	S1 24h		
7	Ctl	ACE2 siRNA	exp2
8	S1 24h		
9	Ctl	Ctl siRNA	
10	S1 24h		
11	Ctl	ACE2 siRNA	exp3
12	S1 24h		

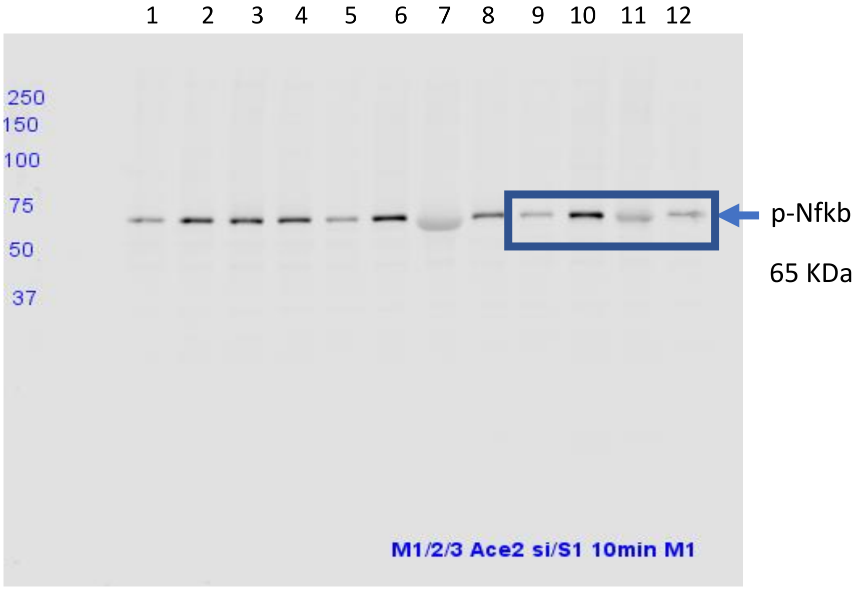

			M1/2/3
1	Ctl	Ctl siRNA	
2	S1 24h		
3	Ctl	ACE2 siRNA	exp1
4	S1 24h		
5	Ctl	Ctl siRNA	
6	S1 24h		
7	Ctl	ACE2 siRNA	exp2
8	S1 24h		
9	Ctl	Ctl siRNA	
10	S1 24h		
11	Ctl	ACE2 siRNA	exp3
12	S1 24h		

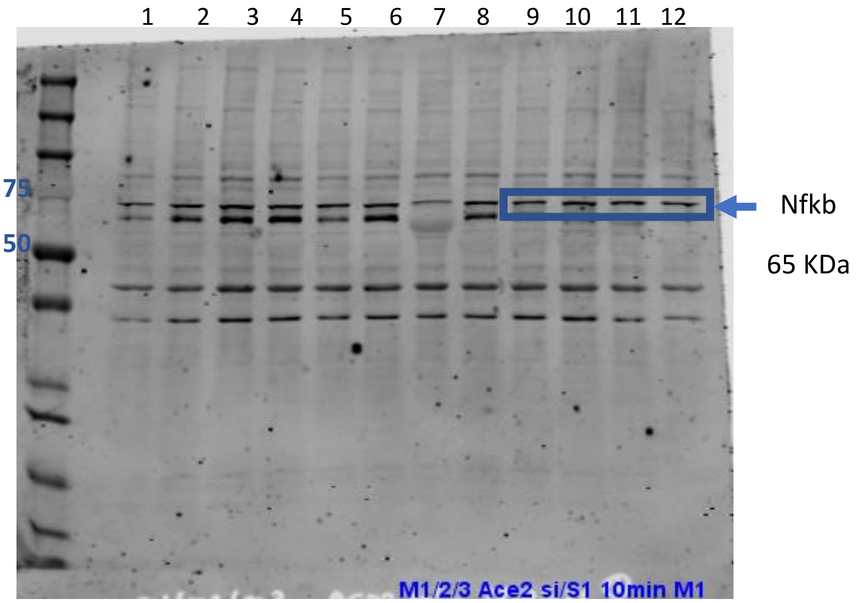

SUPPLEMENTARY FIGURE S20 - ORIGINAL BLOTS – FIGURE 5C – eNOS

			M4/5/6
1	Ctl	Ctl siRNA	
2	S1 24h		
3	Ctl	ACE2 siRNA	exp4
4	S1 24h		
5	Ctl	Ctl siRNA	
6	S1 24h		
7	Ctl	ACE2 siRNA	exp5
8	S1 24h		
9	Ctl	Ctl siRNA	
10	S1 24h		
11	Ctl	ACE2 siRNA	exp6
12	S1 24h		

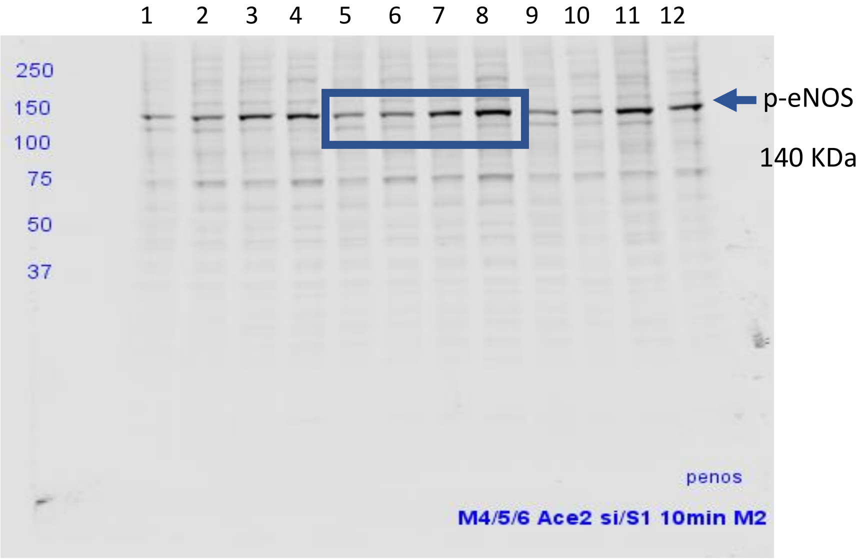

			M4/5/6
1	Ctl	Ctl siRNA	
2	S1 24h		
3	Ctl	ACE2 siRNA	exp4
4	S1 24h		
5	Ctl	Ctl siRNA	
6	S1 24h		
7	Ctl	ACE2 siRNA	exp5
8	S1 24h		
9	Ctl	Ctl siRNA	
10	S1 24h		
11	Ctl	ACE2 siRNA	exp6
12	S1 24h		

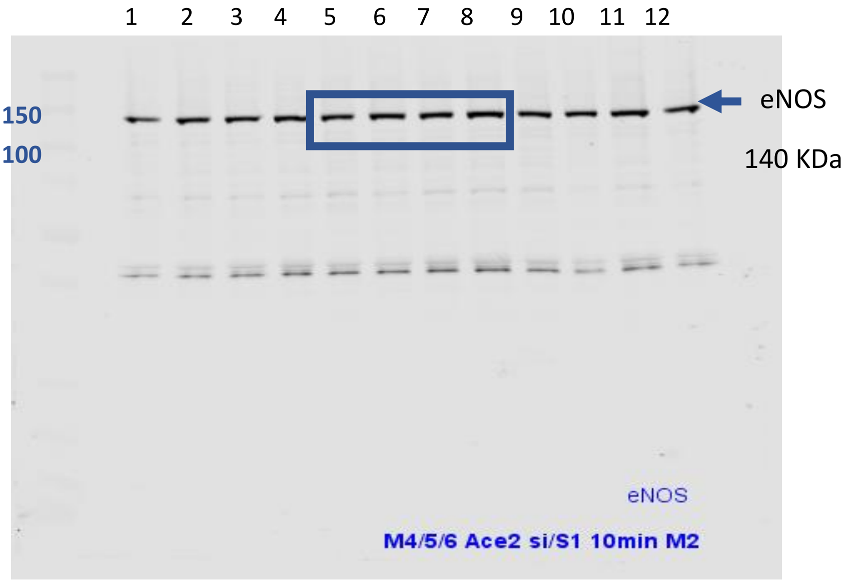

SUPPLEMENTARY FIGURE S21 - ORIGINAL BLOTS – FIGURE 5D – ACE2 expression in Hek293 cells

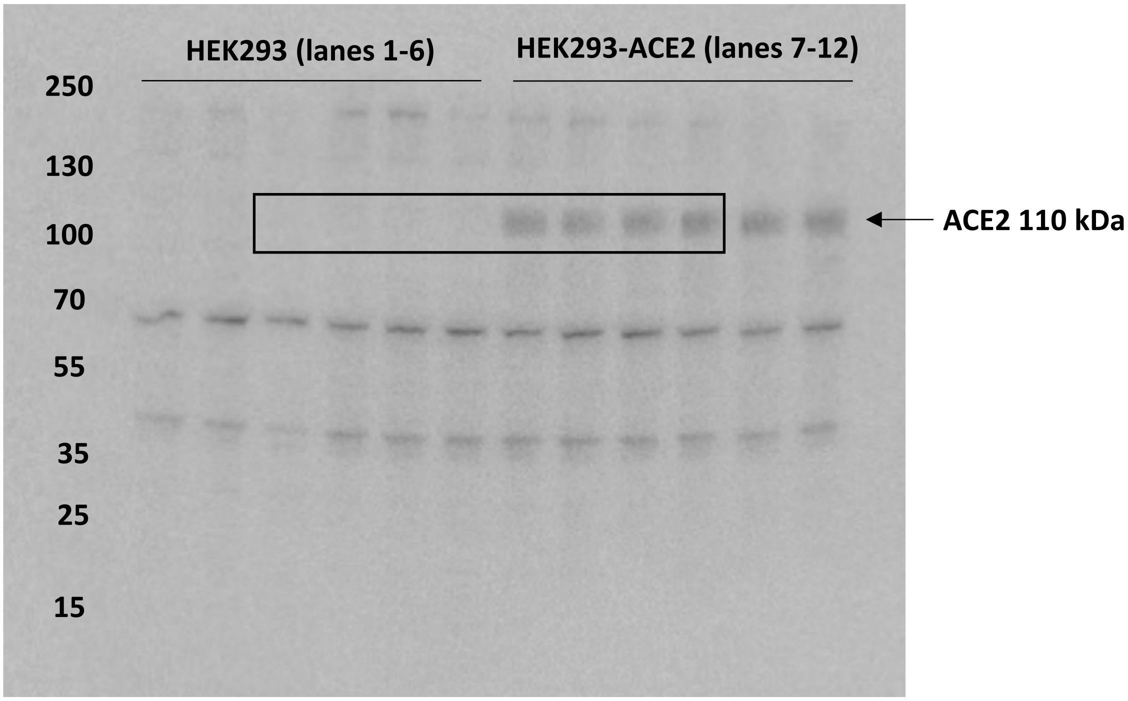

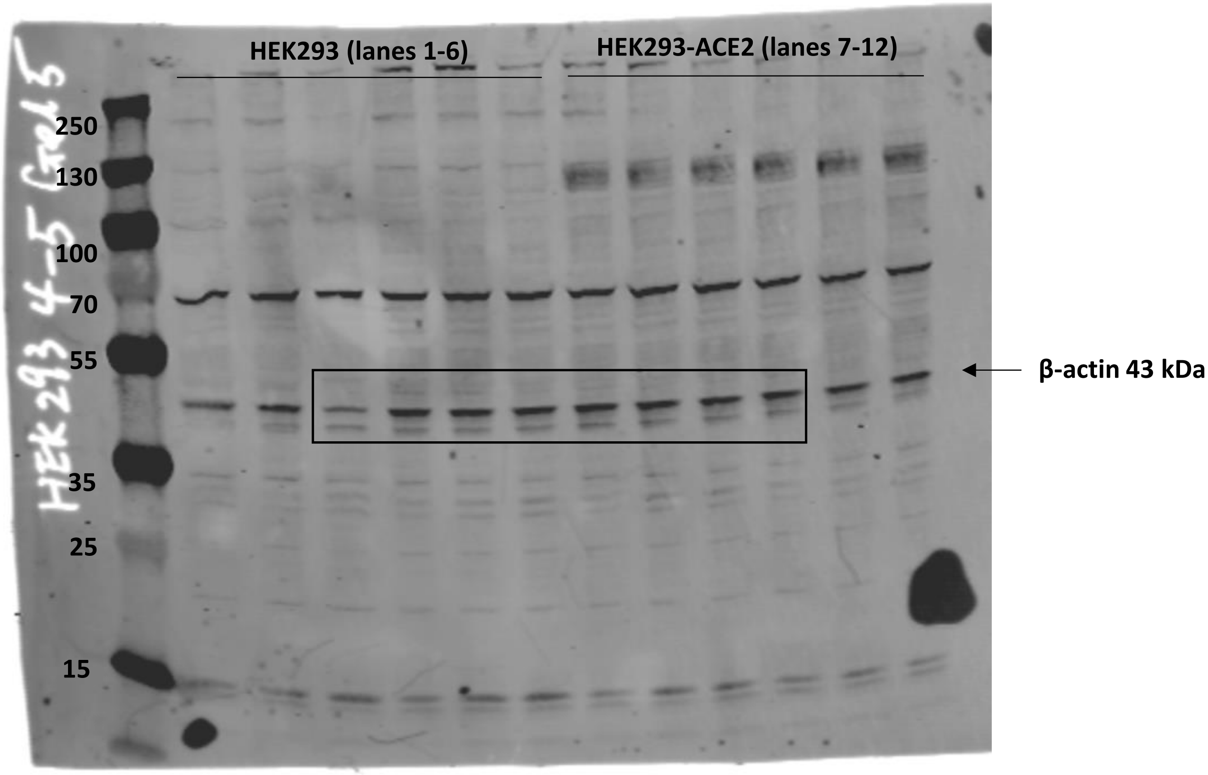

Supplement: Supplementary file 1 — Supplementary Figures. [file 41598_2023_41115_MOESM1_ESM.pdf]
